# Supplementary material for: Increasing the reach of evidence-based interventions for weight management and diabetes prevention among Medicaid patients: study protocol for a pilot Sequential Multiple Assignment Randomised Trial
Source: BMJ Open. 2023 Nov 27;13(11):e075157. doi: 10.1136/bmjopen-2023-075157 (PMC10685946; doi:10.1136/bmjopen-2023-075157)
Supplement: Author's manuscript [file bmjopen-2023-075157.draft_revisions.pdf]

BMJ Open is committed to open peer review. As part of this commitment we make the peer review history of every article we publish publicly available.

When an article is published we post the peer reviewers' comments and the authors' responses online. We also post the versions of the paper that were used during peer review. These are the versions that the peer review comments apply to.

The versions of the paper that follow are the versions that were submitted during the peer review process. They are not the versions of record or the final published versions. They should not be cited or distributed as the published version of this manuscript.

BMJ Open is an open access journal and the full, final, typeset and author-corrected version of record of the manuscript is available on our site with no access controls, subscription charges or pay-per-view fees (http://bmjopen.bmj.com).

If you have any questions on BMJ Open's open peer review process please email info.bmjopen@bmj.com

BMJ Open

Increasing the Reach of Evidence-Based Interventions for Weight Management and Diabetes Prevention Among Medicaid Patients: Study Protocol for a Pilot Sequential Multiple Assignment Randomized Trial.

Journal:	*BMJ Open*
Manuscript ID	bmjopen-2023-075157
Article Type:	Protocol
Date Submitted by the Author:	28-Apr-2023
Complete List of Authors:	Schlechter, Chelsey; University of Utah Health, Population Health Sciences; University of Utah Health Huntsman Cancer Institute<br>Del Fiol, Guilherme; University of Utah, Department of Biomedical Informatics<br>Jones, Dusti; University of Utah Health, Department of Population Health Sciences; University of Utah Health Huntsman Cancer Institute<br>Orleans, Brian; University of Utah Health Huntsman Cancer Institute<br>Gibson, Bryan; University of Utah, Department of Biomedical Informatics<br>Nahum-Shani, Inbal; University of Michigan<br>Maxfield, Ellen; University of Utah Health Care, Osher Center for Integrative Health; University of Utah Health, Department of Family & Preventative Medicine<br>Locke, Amy; University of Utah Health, Osher Center for Integrative Health; University of Utah Health, Department of Family & Preventive Medicine<br>Cornia, Ryan; University of Utah, Department of Biomedical Informatics<br>Bradshaw, Richard; University of Utah, Department of Biomedical Informatics<br>Wirth, Jennifer; University of Utah Health Huntsman Cancer Institute<br>Jaggers, Shanna; University of Utah Health Huntsman Cancer Institute<br>Lam, Cho; University of Utah Health, Department of Population Health Sciences; University of Utah Health Huntsman Cancer Institute<br>Wetter, David; University of Utah Health, Department of Population Health Sciences; University of Utah Health Huntsman Cancer Institute
Keywords:	PUBLIC HEALTH, Obesity, Health Equity, Health informatics < BIOTECHNOLOGY & BIOINFORMATICS, Telemedicine < BIOTECHNOLOGY & BIOINFORMATICS

SCHOLARONE™
Manuscripts

Authors: Chelsey R. Schlechter,[1,2+] Guilherme Del Fiol,[3] Dusti R. Jones,[1,2] Brian Orleans,[2] Bryan Gibson,[3] Inbal Nahum-Shani,[4] Ellen Maxfield,[5,6] Amy Locke,[5,6] Ryan Cornia,[3] Richard Bradshaw,[3] Jennifer Wirth,[2] Shanna J. Jaggers,[2] Cho Y. Lam,[1,2] David W. Wetter[1,2]

[1]Department of Population Health Sciences, University of Utah

[2]Huntsman Cancer Institute, University of Utah

[3]Department of Biomedical Informatics, University of Utah

[4]Institute for Social Research, University of Michigan

[5]Department of Family & Preventative Medicine, University of Utah

[6]Osher Center for Integrative Health, University of Utah

[+] Corresponding Author

Title: Increasing the Reach of Evidence-Based Interventions for Weight Management and Diabetes Prevention Among Medicaid Patients: Study Protocol for a Pilot Sequential Multiple Assignment Randomized Trial.

Abstract

Introduction: Over 40% of United States adults meet criteria for obesity, a major risk factor for cancer, cardiovascular disease, and diabetes. Obesity plays a critical role in health inequities, and disproportionately impacts populations that have been historically marginalized (e.g., low socioeconomic status, rural, some racial/ethnic minority groups). Evidence-based interventions (EBIs) for weight management exist but reach less than 3% of eligible individuals. The aims of this pilot randomized controlled trial are to evaluate feasibility and acceptability of dissemination strategies designed to increase reach of EBIs for weight management.

Methods and analysis: This study is a two-phase, Sequential Multiple Assignment Randomized Trial (SMART), conducted with 200 Medicaid patients. In Phase 1, patients will be individually randomized to single text message (TM1) or multiple text messages (TM+). Phase 2 is based on prior treatment response. Patients who enroll in the EBI within 12 weeks of exposure to Phase 1 (i.e., responders) receive

For peer review only

no further interventions. Patients in TM1 who do not enroll in the EBI within 12 weeks of exposure (i.e., TM1 non-responders) will be randomized to either TM1-Continued (i.e., no further TM) or TM&MAPS (i.e., no further TM, up to 2 Motivation And Problem Solving [MAPS] navigation calls) over the next 12 weeks. Patients in TM+ who do not enroll in the EBI (i.e., TM+ non-responders) will be randomized to either TM+Continued (i.e., monthly text messages) or TM+&MAPS (i.e., monthly text messages, plus up to 2 MAPS calls) over the next 12 weeks. Descriptive statistics will be used to characterize feasibility (e.g., proportion of patients eligible, contacted, and enrolled in the trial) and acceptability (e.g., participant opt-out, participant engagement with dissemination strategies, EBI Reach [i.e., the proportion of participants who enroll in EBI], adherence, effectiveness).

Ethics and dissemination: Study protocol was approved by the University of Utah Institutional Review Board (#00139694).

Strengths and limitations of this study

- Uses an innovative SMART design to optimize adaptive interventions.
- Potential reach and sustainability are enhanced by using dissemination strategy modalities (i.e., cellphone-based voice and text) that are ubiquitous among adults in the United States, including among historically marginalized populations, and are already utilized by payers and healthcare systems.
- Participant population was drawn from a single healthcare system in a single state.

Introduction

Approximately 40% of U.S. adults meet criteria for obesity (BMI ≥30),[1] and this proportion is expected to rise to nearly 50% in the next decade.[2] Obesity is a leading risk factor for multiple chronic diseases, including at least 13 different cancers,[3-5] Type 2 diabetes,[6,7] cardiovascular disease,[8,9] and premature death.[10] Additionally, obesity plays a critical role in health inequities, disproportionately impacting low socioeconomic status (SES), rural, and many racial/ethnic minority populations.[11-15] For example, individuals with lower levels of education have higher obesity rates compared to those with

For peer review only

higher levels of education.[16] Recent studies have found obesity rates of 41% among rural U.S. adults versus 32% among urban residents, and that rising BMI among rural populations has been a principal contributor to the obesity epidemic[17,18] over the last 30 years. Finally, obesity is more prevalent among Latino adults compared to non-Latino White adults (45.6% and 41.4%, respectively).[15]

Evidence-based weight management and diabetes prevention programs. Recommendations from the United States Preventive Services Task Force and others highlight that adults with obesity should engage in comprehensive, multicomponent behavioral interventions addressing weight, diet, and physical activity.[19-22] Components of these behavioral evidence-based interventions (EBIs) include improving diet and nutrition, increasing physical activity, and behavioral strategies such as goal setting and self-monitoring.[23] The Diabetes Prevention Program (DPP) is among the most well-known EBIs for weight management, with substantial evidence for weight loss, increased physical activity, decreased daily energy intake, and decreased incidence of Type 2 diabetes.[24] The DPP can be delivered via in-person or digital modalities, and there are currently over 1800 Centers for Disease Control and Prevention (CDC) recognized DPP programs across the U.S.[25] Despite their effectiveness, DPPs are grossly underutilized,[26] reaching only 3% of eligible individuals.[27] Furthermore, there are substantial disparities in DPP access (14.6% of rural counties contain a DPP site vs. 48.4% for urban counties)[28] and engagement among historically marginalized populations (e.g., low SES, Latino).[26,29] Barriers to participation for in-person delivered DPPs among underserved populations include the need to travel long distances, and the lack of transportation, financial resources, childcare, and time. Fortunately, digital DPP delivery modalities such as the telephone, internet, and smartphones have yielded weight loss and other outcomes comparable to in-person DPPs, with greater engagement and lower attrition in the digital programs.[24,26,30-34] Consistent with those findings, the Community Preventive Services Task Force concluded that "Technology-Supported Multicomponent Coaching or Counseling Interventions" are recommended for both weight loss and weight loss maintenance,[20] and that technology-supported programs may increase access among populations that have transportation issues, unusual work schedules, or are rural. Similarly,

For peer review only

the National Academy of Medicine report on obesity treatment identified key research recommendations that included improving access, employing technology to increase reach, and reducing health disparities.[21]

Dissemination Strategies to Increase EBI Reach. Dissemination strategies are designed to increase the reach of EBIs by increasing individual motivation and capability to access and engage with the interventions.[35,36] Many strategies to increase the reach of weight management EBIs rely on dissemination strategies at the point of care (e.g., provider referral to EBI).[37-39] However, other potent strategies for increasing reach include proactive, population health management approaches that can remove the burden from clinical staff to offer the referral, and can also reach out to all appropriate patients without the need for a clinical visit to trigger the referral offer. Population health management approaches have increased referrals to EBIs for weight management[27,37] and enrollment among eligible participants.[40,41]

Digital and telehealth dissemination strategies such as text messaging and phone-based health coaching are promising approaches to improve dissemination of EBIs for weight management, and have near ubiquitous reach given 97% of Americans own a cell phone, including 97% of individuals with income less than $30,000 per year, 94% of rural individuals, and nearly 100% of Hispanic individuals.[42] Text messaging has been effective for improving numerous health behaviors (e.g., physical activity, nutrition, and tobacco cessation), and for improving attendance and compliance with behavioral interventions.[43-48] Similarly, personalized support to address barriers to care via patient navigation has been shown to improve completion rates for cancer screening, increase adherence to medication, improve compliance to treatment and follow-up visits, improve biomarkers for diabetes, and increase enrollment in EBIs.[49-52] However, there is limited research on the effectiveness of these approaches for increasing reach of EBIs for weight management among historically marginalized populations.

Adaptive interventions. Adaptive interventions contain a sequence of individually tailored decision rules that specify the intensity or type of intervention at critical points in the delivery of care.[53-55]. Modifying intensity or type of intervention over time can improve outcomes if an individual is not

For peer review only

responding, or may reduce costs and burden if intensive treatment is not necessary.[56] For example, a stepped care model (a type of adaptive intervention) can conserve scarce resources and reduce burden on patients. Sequential Multiple Assignment Randomized Trials (SMART[57,58]) are a type of experimental design used to inform the dosage, type, delivery, and timing of adaptive interventions.

Aims and objectives. The overall aims of this pilot study are to evaluate the feasibility and acceptability of the dissemination strategies and study procedures. Feasibility will be measured by the proportion of people who were eligible, contacted, and randomized in the trial. Acceptability will be assessed by rate of participant opt-out, participant engagement with dissemination strategies, EBI Reach by randomization condition, and EBI adherence and effectiveness among participants who enroll in the EBI.

Methods and Analysis

The protocol for this pilot study has been reviewed and approved by the University of Utah Institutional Review Board (IRB). Data collection was planned to begin March 2022 and end in May 2023. The trial is registered at clinicaltrials.gov (NCT05666323). This manuscript adheres to the Standards Protocol Items: Recommendations for Interventional Trials (SPIRIT).

Settings and Participants.

Participants will be 200 adults 1) age ≥ 18, 2) BMI ≥ 30; 2) had a primary care appointment within University of Utah Health (UHealth) within the past 12 months; 3) speak English or Spanish as their primary language; 4) have a current Utah address; and 5) are insured through Medicaid by the University of Utah Health Plans. Exclusion criteria include 1) a diagnosis of Type 1 or Type 2 diabetes, 2) documented current pregnancy, and 3) no phone number listed in the EHR.

Study Design.

This trial is a two-phase, Sequential Multiple Assignment Randomized Trial (SMART) with 200 Medicaid patients from a single academic medical system (Figure 1). In the first phase, participants will be individually randomized to receive a single text message (TM1), or multiple text messages (TM+) offering free enrollment in a digital EBI for weight management.

For peer review only

Phase 2 dissemination strategies are randomized based on prior dissemination strategy response. Participants in TM1 who do not opt out or enroll in EBI by 12 weeks will be randomized to either TM1-Continued (no further TM) or TM1 & MAPS (no further TM and up to 2 Motivation And Problem Solving [MAPS] calls from a patient navigator). Participants in TM+ who do not opt out or enroll in the EBI by 12 weeks will be randomized to either TM+ Continued (monthly text messages for the next 12 weeks) or TM+ & MAPS (monthly text messages for the next 12 weeks, plus up to 2 MAPS calls from a patient navigator). Descriptions of participant interventions are listed in Table 1.

Table 1: Randomization Condition Description

	Randomization Condition	Description
**Phase 1**		
	Single text message (TM1)	Participants receive a single text message that includes a motivational message, the EBI phone number, and a two-touch response that directly connects interested participants to EBI enrollment.
	Multiple text message (TM+)	Participants receive messages once per week for the first 2 weeks, then 1 message every 3 weeks for the remaining 10 weeks (up to 5 texts total). The text messages include a motivational message, the EBI phone number, and a simple two-touch response that directly connects interested participants to EBI enrollment.
**Phase 2**		
For TM1 non-responding participants only.	Single text message continued (TM1-Cont)	Participants receive no additional text messages.
	Single text message & Motivation And Problem Solving (MAPS) (TM1 & MAPS)	Participants receive no additional text messages and receive up to 2 MAPS counselling calls from a health coach.
For TM+ non-responding participants only.	Multiple text message continued (TM+-Cont)	Participants receive text messages every 4 weeks for the next 12 weeks (up to 3 text messages total).
	Multiple text message & MAPS (TM+ & MAPS)	Participants receive text messages every 4 weeks for the next 12 weeks (up to 3 text messages total) plus up to 2 MAPS counselling calls from a health coach.

Dissemination Strategies

Text Messaging. Participants will receive bidirectional Health Insurance Portability and Accountability Act (HIPAA) compliant text messages that include a brief motivational message, the

For peer review only

opportunity to enroll in the EBI, and the opportunity to opt-out of receiving further dissemination strategies.

Motivation And Problem Solving (MAPS) Health Coaching. Participants will receive up to two brief calls from health coaches trained in using MAPS, a coaching/counselling approach designed to help participants address the larger context in which behavior change occurs, identify and address barriers to change, and motivate participants towards a long-term goal of change. MAPS is a holistic, dynamic behavioral approach that integrates two empirically validated approaches (motivational interviewing[59,60] and problem solving/social cognitive strategies[61-63]) into a coordinated intervention with demonstrated efficacy for addressing the entire process of considering behavior change, attempting to change, and maintaining change.[50,63-66] MAPS employs a "wellness" approach that in addition to focusing on the target behavior, provides practical advice and connections to services whenever possible for those life events, stressors, and concerns that impact the lives of rural and other underserved individuals (e.g., isolation, stress, depression, financial barriers, etc.,). MAPS is based on substantial evidence that motivation fluctuates frequently and rapidly, and as such, is designed to fluidly respond to those changes.[67-70] MAPS health coaching calls will be delivered by two bilingual health coaches on the research team with at least 20 hours of training in MAPS delivery.

Digital EBI for Diabetes Prevention and Weight Management.

Participants who enroll in the EBI will receive either a digital DPP or digital weight management program based on criteria for meeting prediabetes. Participants will be evaluated for risk of prediabetes using the American Diabetes Association (ADA) and CDC Prediabetes Risk Test[71] at the time of enrolling in the EBI. The test uses only self-report health trait data (e.g., family history, height, weight, physical activity level, health history of hypertension and gestational diabetes) and does not require testing data (i.e., hemoglobin A1C or cholesterol levels). Participants who meet criteria for prediabetes based on the risk test will be triaged into a digital DPP program; participants who do not meet criteria will be triaged to a digital weight management program (both programs delivered by EBI vendor,

For peer review only

IncentaHEALTH). Based on national data on eligibility for the DPP among individuals with obesity and data from the EBI provider, we estimate that ~50% of participants will be triaged to DPP and ~50% to weight management.[72]

The digital DPP offered to study participants is a CDC fully recognized program. The digital weight management program, for all participants who do not meet criteria for the DPP, uses the same general components as the DPP. Both programs are designed to increase intake of fruits, vegetables, lean protein and complex carbohydrates while monitoring portion size. Physical activity recommendations include cardiovascular and strength training, with a goal of approximately 30 minutes 5-6 days/week. Both programs are offered in English and Spanish, based on Social Cognitive Theory, and provide personalized health coaching. Both include a website, a Bluetooth wireless scale for weight monitoring, daily email/text message support, easy to follow video exercise plans, customized meal plans, grocery lists, and incorporate skill building, and social accountability. Participants have unlimited access to Health Coaches, who have at least a Bachelor's degree, along with certifications, in personal training, nutrition, or wellness.

Recruitment.

The flow of participants through the study is described in Figure 2. Participants who meet inclusion criteria will be identified from the University of Utah Health (UHealth) Electronic Data Warehouse (EDW). Participant demographic, preferred language, and relevant health data, as well as participant contact information (i.e., phone number, address, email address) will be extracted from the EDW. Participants who meet exclusionary criteria will be removed from the eligible population. We will use quota sampling from the eligible population to achieve a stratified random sample aimed to offer balance in the marginal distributions of key participant demographic characteristics. These margins include 1) male/female sex (1:1 ratio), 2) urban/rural according to participant zip code and the 2010 Rural Urban Commuting Area (RUCA) codes (>= 4 defined as rural) (1:1 ratio), and 3) Latino with English as preferred language, Latino with Spanish as preferred language, and non-Latino with English as preferred language (1:1:3 ratio).

For peer review only

Sampled patients randomized to TM1 or TM+ will be sent an e-mail informing them that they will be receiving communications from UHealth about the availability of a digital EBI for weight management. The e-mail will provide patients with information about how they can opt-out if they do not wish to receive these communications. Patients who do not have an email in the EHR will be sent the opt-out information via mail. Patients who opt-out of the study receive no further interventions.

Randomization and Blinding.

In Phase 1 (Figure 1), participants will be randomly assigned to TM1 or TM+ in a 1:1 ratio. In Phase 2 (Figure 1), participants who opt out or who enroll in the EBI will receive no further dissemination strategies and will not be re-randomized. Participants in TM1 did not opt-out and who did not enroll in the EBI will be randomized to TM1-Cont or TM1 & MAPS at a ratio of 1:1. Participants in TM+ who did not opt-out and did not enroll in the EBI will be randomized to TM+-Cont or TM+ & MAPS at a ratio of 1:1. All randomization sequences will be generated via SAS 9.4 to consist of random permuted blocks with random block sizes, thus ensuring balance in the allocation over time.

Data Collection and Outcomes.

Participant demographic and baseline health data (e.g., age, gender, BMI, pregnancy status, medical codes for Type 1 and Type 2 diabetes, zip code, preferred language, race/ethnicity) will be obtained from the UHealth EDW.

Feasibility outcomes. Feasibility will be assessed using the proportion of participants who were eligible, contacted, and enrolled in the trial.

Acceptability outcomes. Acceptability will be assessed using participant opt-out, participant engagement with dissemination strategies, EBI reach, and EBI effectiveness. Participant opt-out is assessed by the absolute number and proportion of eligible participants who opt-out during Phase 1 and Phase 2. Participant engagement will be assessed as 1) the number and proportion of participants who respond to text messages with a response other than opt-out, 2) the number and proportion of eligible participants who talk to a MAPS health coach, 3) the number and proportion of eligible participants accept connection to the EBI, and 4) the number and proportion of participants who talk to the EBI

For peer review only

personnel. EBI reach is assessed by the number and proportion of eligible participants who officially enroll in the EBI. Enrollment is defined in accordance with the CDC definition for recognized virtual programs, which consists of the participant setting a password for the program. Enrollment will be obtained from the EBI vendor (IncentaHEALTH). EBI adherence and effectiveness are measured by participant adherence to the EBI components, percentage of body-weight lost, and change in BMI, and will be collected for 12 weeks following EBI enrollment. Adherence and effectiveness data will be obtained from the EBI vendor (IncentaHEALTH)

Patient and Public Involvement.

This study was developed and implemented in collaboration with the University of Utah Wellness and Integrative Health Program, which supports population health initiatives through integration of wellness services into patient care and health plan offerings. The program currently manages lifestyle behavior change programs (including the Diabetes Prevention Program) for UHealth patients and University of Utah faculty, staff, and students. The dissemination strategies, the feasibility and acceptability outcomes, and study procedures were developed in collaboration with the University of Utah Wellness and Integrative Health Program and investigators at the University of Utah. Patients were not directly involved in the development of this pilot study.

Ethics and dissemination.

All procedures performed in studies involving human participants will be conducted in accordance with the ethical standards of the institutional and/or national research committee and with the 1964 Helsinki declaration and its later amendments or comparable ethical standards. The protocol for this study was approved by the University of Utah Institutional Review Board (00139694). Materials used to conduct the study are not currently publicly available. Materials may be requested by emailing the corresponding author. Study results will be dissemination via peer reviewed publications and manuscripts, as well as to the health system and community partners via lay reports and presentations.

Data Analysis Plan.

For peer review only

Our primary study aims to evaluate the acceptability and feasibility of dissemination strategies to increase of EBIs for weight management.

Primary Analyses Phase 1. The analysis of acceptability and feasibility at 12 weeks will include all participants enrolled in the study. We will use descriptive statistics (mean, standard deviation (SD), confidence interval) to evaluate each indicator of feasibility and acceptability by dissemination strategies (i.e., TM1, TM+).

Primary Analyses Phase 2. The analysis of feasibility and acceptability at 24-weeks will be conducted using only data from those participants who did not opt out or enroll in the EBIs by 12 weeks (i.e., non-responders). We will use descriptive statistics to evaluate each indicator of feasibility and acceptability by dissemination strategies (i.e., TM1-Cont, TM1 & MAPS, TM+-Cont, TM+ & MAPS).

Power Analysis.

The sample size (N = 200) for the current project was determined based on feasibility of sample size recruitment from a single Medicaid provider and completing project procedures in the study timeframe. As such, power calculations were not conducted for this pilot trial.

Conclusion

The proposed pilot project is a two-phase SMART with the objective of evaluating acceptability and feasibility of dissemination strategies designed to increase the reach of digital EBIs for weight management among Medicaid patients. Accomplishing the aims of the proposed study will provide critical data regarding the feasibility and acceptability of pragmatic and scalable dissemination strategies designed to increase the uptake of digital EBIs for obesity among historically marginalized populations, and inform the conduct of a future, fully powered randomized trial.

Registration details: The trial is registered at clinicaltrials.gov (NCT05666323).

Funding statement. This work was supported through the Ben B. and Iris M. Margolis Foundation, the National Center for Advancing Translational Sciences of the National Institutes of Health (UL1TR002538), the National Cancer Institute (P30CA042014), and the Huntsman Cancer Foundation.

For peer review only - http://bmjopen.bmj.com/site/about/guidelines.xhtml

Competing interests. The authors declare no competing interests.

Author contribution. DWW acquired financial support for the project. CRS, GDF, BO, BG, EM, AL, JW, CYL, and DWW contributed to study conceptualization. All authors contributed to the development or design of the methodology, dissemination strategies, study procedures, study outcomes, or analytic plan. CRS and DRJ drafted the original manuscript. All authors reviewed, edited, and approved the final manuscript.

References

1. Hales CM, Carroll MD, Fryar CD, Ogden CL. Prevalence of Obesity and Severe Obesity Among Adults: United States, 2017–2018. *NCHS Data Brief.* 2020;no. 360(1-7).
2. Ward ZJ, Bleich SN, Cradock AL, et al. Projected U.S. State-Level Prevalence of Adult Obesity and Severe Obesity. *N Engl J Med.* 2019;381(25):2440-2450.
3. Islami F, Goding Sauer A, Miller KD, et al. Proportion and number of cancer cases and deaths attributable to potentially modifiable risk factors in the United States. *CA: A Cancer Journal for Clinicians.* 2018;68(1):31-54.
4. Islami F, Goding Sauer A, Gapstur SM, Jemal A. Proportion of Cancer Cases Attributable to Excess Body Weight by US State, 2011-2015. *JAMA Oncology.* 2019;5(3):384-392.
5. McTiernan A, Friedenreich CM, Katzmarzyk PT, et al. Physical Activity in Cancer Prevention and Survival: A Systematic Review. *Med Sci Sports Exerc.* 2019;51(6):1252-1261.
6. Verma S, Hussain ME. Obesity and diabetes: An update. *Diabetes Metab Syndr.* 2017;11(1):73-79.
7. Mokdad AH, Ford ES, Bowman BA, et al. Prevalence of obesity, diabetes, and obesity-related health risk factors, 2001. *Jama.* 2003;289(1):76-79.
8. Powell-Wiley TM, Poirier P, Burke LE, et al. Obesity and cardiovascular disease: a scientific statement from the American Heart Association. *Circulation.* 2021;143(21):e984-e1010.
9. Khan SS, Ning H, Wilkins JT, et al. Association of Body Mass Index With Lifetime Risk of Cardiovascular Disease and Compression of Morbidity. *JAMA Cardiol.* 2018;3(4):280-287.
10. Hruby A, Manson JE, Qi L, et al. Determinants and Consequences of Obesity. *Am J Public Health.* 2016;106(9):1656-1662.
11. AHRQ. *2018 National Healthcare Quality and Disparities.* Rockville, MD: Agency for Healthcare Research and Quality;September 2019.
12. Clarke AR, Goddu AP, Nocon RS, et al. Thirty years of disparities intervention research: what are we doing to close racial and ethnic gaps in health care? *Med Care.* 2013;51(11):1020-1026.
13. Crosby RA, Wendel ML, Vanderpool RC, Casey BR, eds. *Rural populations and health: determinants, disparities, and solutions.* San Francisco, CA: Jossey-Bass; 2012.

For peer review only

14. National Center for Health Statistics. *Health, United States, 2011: With Special Feature on Socioeconomic Status and Health.* Hyattsville, MD: National Center for Health Statistics (US); 2012.
15. Akinbami LJ, Chen TC, Davy O, et al. National Health and Nutrition Examination Survey, 2017-March 2020 Prepandemic File: Sample Design, Estimation, and Analytic Guidelines. *Vital Health Stat 1.* 2022(190):1-36.
16. Stierman B, Afful J, Carroll MD, et al. National Health and Nutrition Examination Survey 2017–March 2020 prepandemic data files development of files and prevalence estimates for selected health outcomes. 2021.
17. NCD Risk Factor Collaboration. Rising rural body-mass index is the main driver of the global obesity epidemic in adults. *Nature.* 2019;569(7755):260.
18. Wen M, Fan JX, Kowaleski-Jones L, Wan N. Rural–urban disparities in obesity prevalence among working age adults in the United States: exploring the mechanisms. *American Journal of Health Promotion.* 2018;32(2):400-408.
19. Jensen MD, Ryan DH, Apovian CM, et al. 2013 AHA/ACC/TOS guideline for the management of overweight and obesity in adults: a report of the American College of Cardiology/American Heart Association task force on practice guidelines and the obesity society. *Circulation.* 2014;129(25 Suppl 2):S102-S138.
20. Community Preventive Services Task Force. *Obesity: Technology-Supported Multicomponent Coaching or Counseling Interventions to Maintain Weight Loss. Retrieved from https://www.thecommunityguide.org/findings/obesity-technology-supported-multicomponent-coaching-or-counseling-interventions-maintain.*
21. Heymsfield S, Aronne LJ, Eneli I, et al. Clinical Perspectives on Obesity Treatment: Challenges, Gaps, and Promising Opportunities. *NAM Perspectives.* 2018.
22. LeBlanc ES, Patnode CD, Webber EM, Redmond N, Rushkin M, O'Connor EA. Behavioral and pharmacotherapy weight loss interventions to prevent obesity-related morbidity and mortality in adults: Updated evidence report and systematic review for the US Preventive Services Task Force. *Jama.* 2018;320(11):1172-1191.
23. Curry SJ, Krist AH, Owens DK, et al. Behavioral weight loss interventions to prevent obesity-related morbidity and mortality in adults: US preventive services task force recommendation statement. *Jama.* 2018;320(11):1163-1171.
24. Dunkley AJ, Bodicoat DH, Greaves CJ, et al. Diabetes prevention in the real world: effectiveness of pragmatic lifestyle interventions for the prevention of type 2 diabetes and of the impact of adherence to guideline recommendations: a systematic review and meta-analysis. *Diabetes care.* 2014;37(4):922-933.
25. CDC. Diabetes Prevention Recognition Program – Registry of Recognized Organizations. https://nccd.cdc.gov/DDT_DPRP/Registry.aspx. Accessed 05/14/2020.
26. Ali MK, Bullard KM, Imperatore G, et al. Reach and use of diabetes prevention services in the United States, 2016-2017. *JAMA network open.* 2019;2(5):e193160-e193160.
27. Alva ML, Chakkalakal RJ, Moin T, Galaviz KI. The Diabetes Prevention Gap And Opportunities To Increase Participation In Effective Interventions. *Health Aff (Millwood).* 2022;41(7):971-979.
28. Ariel-Donges AH, Gordon EL, Dixon BN, et al. Rural/urban disparities in access to the National Diabetes Prevention Program. *Translational behavioral medicine.* 2019.

For peer review only

29. Ritchie ND, Christoe-Frazier L, McFann KK, Havranek EP, Pereira RI. Effect of the National Diabetes Prevention Program on Weight Loss for English- and Spanish-Speaking Latinos. *American Journal of Health Promotion.* 2017;32(3):812-815.
30. Moin T, Damschroder LJ, AuYoung M, et al. Results From a Trial of an Online Diabetes Prevention Program Intervention. *American Journal of Preventive Medicine.* 2018;55(5):583-591.
31. Mudaliar U, Zabetian A, Goodman M, et al. Cardiometabolic risk factor changes observed in diabetes prevention programs in US settings: a systematic review and meta-analysis. *PLoS medicine.* 2016;13(7).
32. Taetzsch A, Gilhooly CH, Bukhari A, et al. Development of a Videoconference-Adapted Version of the Community Diabetes Prevention Program, and Comparison of Weight Loss With In-Person Program Delivery. *Military Medicine.* 2019;184(11-12):647-652.
33. Tice J, Chapman R, Shore KK, et al. Diabetes Prevention Programs: effectiveness and value: final evidence report and meeting summary. 2018.
34. Kim SE, Sweet CMC, Gibson E, et al. Evaluation of a digital diabetes prevention program adapted for the Medicaid population: study design and methods for a non-randomized, controlled trial. *Contemporary clinical trials communications.* 2018;10:161-168.
35. Lomas J. Diffusion, dissemination, and implementation: who should do what? *Ann N Y Acad Sci.* 1993;703:226-235; discussion 235-227.
36. McCormack L, Sheridan S, Lewis M, et al. Communication and dissemination strategies to facilitate the use of health-related evidence. *Evid Rep Technol Assess (Full Rep).* 2013(213):1-520.
37. Holliday CS, Williams J, Salcedo V, Kandula NR. Clinical Identification and Referral of Adults With Prediabetes to a Diabetes Prevention Program. *Prev Chronic Dis.* 2019;16:E82.
38. Nhim K, Khan T, Gruss S, et al. Facilitators to referrals to CDC's National Diabetes Prevention Program in primary care practices and pharmacies: DocStyles 2016-2017. *Prev Med.* 2021;149:106614.
39. Kirley K, Khan T, Aquino G, et al. Using a certified electronic health record technology platform to screen, test and refer patients with prediabetes. *JAMIA Open.* 2021;4(4):ooab101.
40. Porter G, Michaud TL, Schwab RJ, Hill JL, Estabrooks PA. Reach Outcomes and Costs of Different Physician Referral Strategies for a Weight Management Program Among Rural Primary Care Patients: Type 3 Hybrid Effectiveness-Implementation Trial. *JMIR Formative Research.* 2021;5(10):e28622.
41. Wilson KE, Michaud TL, Almeida FA, et al. Using a population health management approach to enroll participants in a diabetes prevention trial: reach outcomes from the PREDICTS randomized clinical trial. *Translational behavioral medicine.* 2021;11(5):1066-1077.
42. Center PR. Mobile Fact Sheet. https://www.pewresearch.org/internet/fact-sheet/mobile/. Published 2021. Accessed 3/15/2021.
43. Cole-Lewis H, Kershaw T. Text messaging as a tool for behavior change in disease prevention and management. *Epidemiologic reviews.* 2010:mxq004.

For peer review only

44. Gurol-Urganci I, de Jongh T, Vodopivec-Jamsek V, Atun R, Car J. Mobile phone messaging reminders for attendance at healthcare appointments. *Cochrane Database of Systematic Reviews.* 2013(12).
45. Head KJ, Noar SM, Iannarino NT, Harrington NG. Efficacy of text messaging-based interventions for health promotion: a meta-analysis. *Social Science & Medicine.* 2013;97:41-48.
46. Schwebel FJ, Larimer ME. Using text message reminders in health care services: A narrative literature review. *Internet Interventions.* 2018;13:82-104.
47. Vodopivec-Jamsek V, de Jongh T, Gurol-Urganci I, Atun R, Car J. Mobile phone messaging for preventive health care. *The Cochrane Library.* 2012.
48. USDHHS. *Using Health Text Messages To Improve Consumer Health Knowledge, Behaviors, And Outcomes: An Environmental Scan.* Rockville, MD: US Department of Health and Human Services;2014.
49. McBrien KA, Ivers N, Barnieh L, et al. Patient navigators for people with chronic disease: A systematic review. *PloS one.* 2018;13(2):e0191980-e0191980.
50. Vinci C, Lam C, Schlechter CR, Shono Y, Vidrine JI, Wetter DW. Increasing treatment enrollment among smokers who are not motivated to quit: a randomized clinical trial. *Translational Behavioral Medicine.* 2021.
51. Peart A, Lewis V, Brown T, Russell G. Patient navigators facilitating access to primary care: a scoping review. *BMJ open.* 2018;8(3):e019252.
52. Valaitis RK, Carter N, Lam A, Nicholl J, Feather J, Cleghorn L. Implementation and maintenance of patient navigation programs linking primary care with community-based health and social services: a scoping literature review. *BMC health services research.* 2017;17(1):116.
53. Almirall D, Collins LM, Murphy SA. Introduction to adaptive interventions and SMART study design principles. COPTR Workshop on Adaptive Interventions 2011.
54. Bierman KL, Nix RL, Maples JJ, Murphy SA. Examining clinical judgment in an adaptive intervention design: The fast track program. *Journal of Consulting and Clinical Psychology.* 2006;74(3):468.
55. McKay JR. Is there a case for extended interventions for alcohol and drug use disorders? *Addiction.* 2005;100(11):1594-1610.
56. Lei H, Nahum-Shani I, Lynch K, Oslin D, Murphy S. A" SMART" design for building individualized treatment sequences. *Annual review of clinical psychology.* 2012;8:21-48.
57. Lavori PW, Dawson R. Introduction to dynamic treatment strategies and sequential multiple assignment randomization. *Clinical Trials.* 2014:1740774514527651.
58. Murphy SA. An experimental design for the development of adaptive treatment strategies. *Statistics in medicine.* 2005;24(10):1455-1481.
59. Miller WR, Rollnick S. *Motivational Interviewing: Preparing People to Change Addictive Behavior.* New York: The Guildford Press; 1991.
60. Miller WR, Rollnick S. *Motivational Interviewing (2nd edition).* New York: The Guilford Press; 2002.
61. Marlatt G, Donovan D, eds. *Relapse prevention: Maintenance strategies in the treatment of addictive behaviors (2nd edition).* New York: Guilford Press; 2005.
62. Witkiewitz K, Marlatt GA. Relapse prevention for alcohol and drug problems: That was Zen, this is Tao. *American Psychologist.* 2004;59(4):224-235.

For peer review only

63. Reitzel LR, Vidrine JI, Businelle MS, et al. Preventing postpartum smoking relapse among diverse low-income women: a randomized clinical trial. *Nicotine Tob Res.* 2010;12(4):326-335.
64. Correa-Fernández V, Díaz-Toro EC, Reitzel LR, et al. Combined treatment for at-risk drinking and smoking cessation among Puerto Ricans: A randomized clinical trial. *Addictive Behaviors.* 2017;65:185-192.
65. McClure JB, Westbrook E, Curry SJ, Wetter DW. Proactive, motivationally enhanced smoking cessation counseling among women with elevated cervical cancer risk. *Nicotine & Tobacco Research.* 2005;7(6):881-889.
66. Wetter DW, Mazas C, Daza P, et al. Reaching and treating Spanish-speaking smokers through the National Cancer Institute's Cancer Information Service. A randomized controlled trial. *Cancer.* 2007;109(2 Suppl):406-413.
67. Conroy DE, Elavsky S, Hyde AL, Doerksen SE. The dynamic nature of physical activity intentions: a within-person perspective on intention-behavior coupling. *Journal of Sport and Exercise Psychology.* 2011;33(6):807-827.
68. Maher JP, Dunton GF. Within-day time-varying associations between motivation and movement-related behaviors in older adults. *Psychology of Sport and Exercise.* 2020;47:101522.
69. Vartanian LR, Pinkus RT, Smyth JM. Experiences of weight stigma in everyday life: Implications for health motivation. *Stigma and Health.* 2018;3(2):85-92.
70. Vidrine JI, Reitzel LR, Figueroa P, et al. Motivation And Problem Solving (MAPS): Motivationally-based skills training for treating substance use. *Cogn Behav Pract.* 2013;20(4):501-516.
71. Centers for Disease Control Prevention ADA. Predaibetes Risk Test. https://www.cdc.gov/diabetes/prevention/pdf/Prediabetes-Risk-Test-Final.pdf. Accessed January 3, 2023.
72. Bullard KM, Saydah SH, Imperatore G, et al. Secular changes in US prediabetes prevalence defined by hemoglobin A1c and fasting plasma glucose: National Health and Nutrition Examination Surveys, 1999–2010. *Diabetes Care.* 2013;36(8):2286-2293.
73. Nahum-Shani I, Qian M, Almirall D, et al. Experimental design and primary data analysis methods for comparing adaptive interventions. *Psychol Methods.* 2012;17(4):457-477.

For peer review only

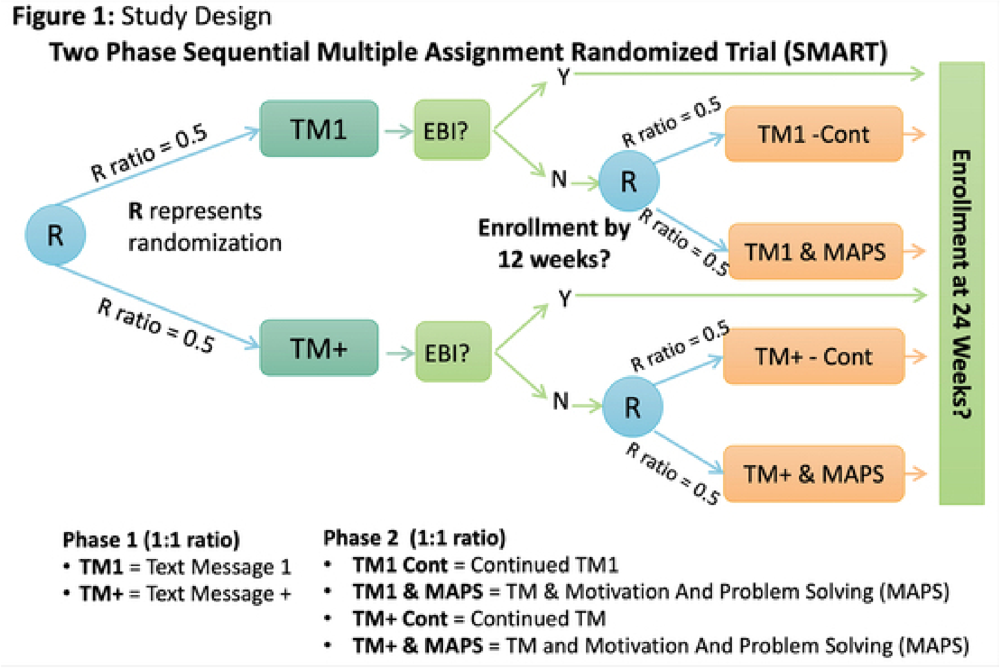

Figure 1: Study Design

195x130mm (72 x 72 DPI)

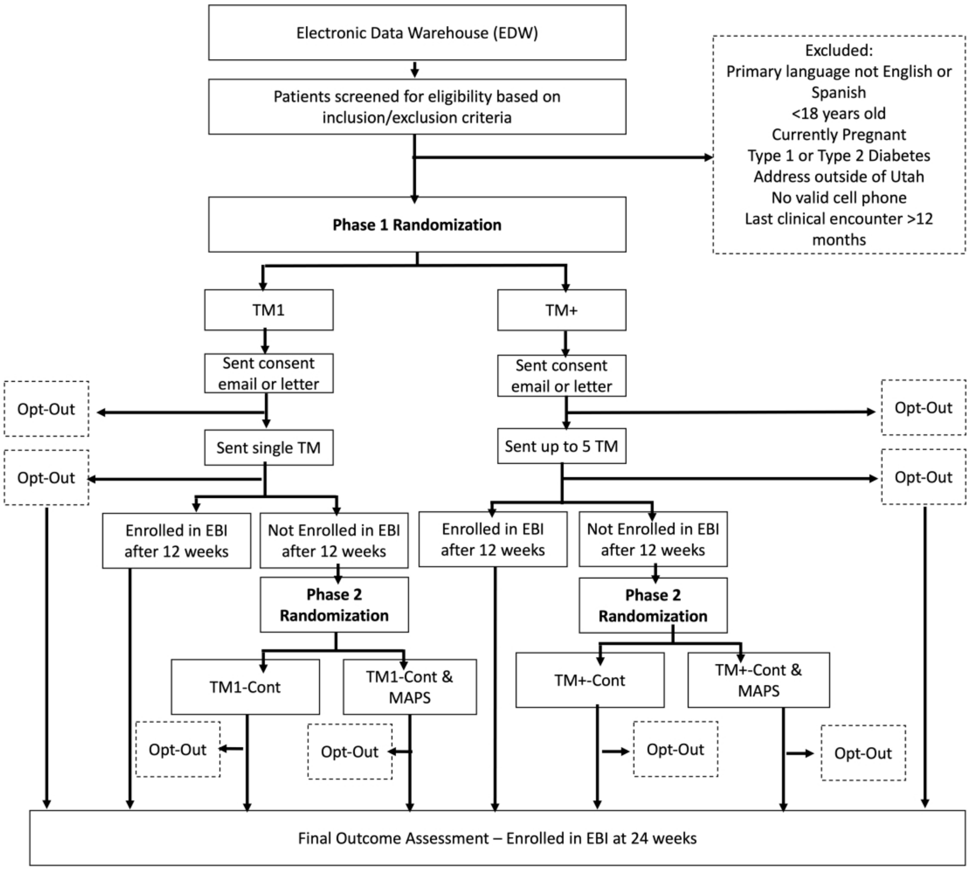

Figure 2: Consort Diagram

361x321mm (300 x 300 DPI)

SPIRIT

STANDARD PROTOCOL ITEMS: RECOMMENDATIONS FOR INTERVENTIONAL TRIALS

SPIRIT 2013 Checklist: Recommended items to address in a clinical trial protocol and related documents*

	Section/item	ItemNo	Description
	**Administrative information**		
Y	Title	1	Descriptive title identifying the study design, population, interventions, and, if applicable, trial acronym
Y	Trial registration	2a	Trial identifier and registry name. If not yet registered, name of intended registry
NA		2b	All items from the World Health Organization Trial Registration Data Set
NA	Protocol version	3	Date and version identifier
Y	Funding	4	Sources and types of financial, material, and other support
Y	Roles and responsibilities	5a	Names, affiliations, and roles of protocol contributors
Y		5b	Name and contact information for the trial sponsor
Y		5c	Role of study sponsor and funders, if any, in study design; collection, management, analysis, and interpretation of data; writing of the report; and the decision to submit the report for publication, including whether they will have ultimate authority over any of these activities
NA		5d	Composition, roles, and responsibilities of the coordinating centre, steering committee, endpoint adjudication committee, data management team, and other individuals or groups overseeing the trial, if applicable (see Item 21a for data monitoring committee)
	**Introduction**		
Y	Background and rationale	6a	Description of research question and justification for undertaking the trial, including summary of relevant studies (published and unpublished) examining benefits and harms for each intervention

For peer review only

Y		6b	Explanation for choice of comparators
Y	Objectives	7	Specific objectives or hypotheses
Y	Trial design	8	Description of trial design including type of trial (eg, parallel group, crossover, factorial, single group), allocation ratio, and framework (eg, superiority, equivalence, noninferiority, exploratory)
	**Methods: Participants, interventions, and outcomes**		
Y	Study setting	9	Description of study settings (eg, community clinic, academic hospital) and list of countries where data will be collected. Reference to where list of study sites can be obtained
Y	Eligibility criteria	10	Inclusion and exclusion criteria for participants. If applicable, eligibility criteria for study centres and individuals who will perform the interventions (eg, surgeons, psychotherapists)
Y	Interventions	11a	Interventions for each group with sufficient detail to allow replication, including how and when they will be administered
NA		11b	Criteria for discontinuing or modifying allocated interventions for a given trial participant (eg, drug dose change in response to harms, participant request, or improving/worsening disease)
NA		11c	Strategies to improve adherence to intervention protocols, and any procedures for monitoring adherence (eg, drug tablet return, laboratory tests)
NA		11d	Relevant concomitant care and interventions that are permitted or prohibited during the trial
Y	Outcomes	12	Primary, secondary, and other outcomes, including the specific measurement variable (eg, systolic blood pressure), analysis metric (eg, change from baseline, final value, time to event), method of aggregation (eg, median, proportion), and time point for each outcome. Explanation of the clinical relevance of chosen efficacy and harm outcomes is strongly recommended
Y	Participant timeline	13	Time schedule of enrolment, interventions (including any run-ins and washouts), assessments, and visits for participants. A schematic diagram is highly recommended (see Figure)

For peer review only - http://bmjopen.bmj.com/site/about/guidelines.xhtml

Y	Sample size	14	Estimated number of participants needed to achieve study objectives and how it was determined, including clinical and statistical assumptions supporting any sample size calculations
Y	Recruitment	15	Strategies for achieving adequate participant enrolment to reach target sample size
	**Methods: Assignment of interventions (for controlled trials)**		
	Allocation:		
Y	Sequence generation	16a	Method of generating the allocation sequence (eg, computer-generated random numbers), and list of any factors for stratification. To reduce predictability of a random sequence, details of any planned restriction (eg, blocking) should be provided in a separate document that is unavailable to those who enrol participants or assign interventions
NA	Allocation concealment mechanism	16b	Mechanism of implementing the allocation sequence (eg, central telephone; sequentially numbered, opaque, sealed envelopes), describing any steps to conceal the sequence until interventions are assigned
Y	Implementation	16c	Who will generate the allocation sequence, who will enrol participants, and who will assign participants to interventions
NA	Blinding (masking)	17a	Who will be blinded after assignment to interventions (eg, trial participants, care providers, outcome assessors, data analysts), and how
NA		17b	If blinded, circumstances under which unblinding is permissible, and procedure for revealing a participant's allocated intervention during the trial
	**Methods: Data collection, management, and analysis**		
Y	Data collection methods	18a	Plans for assessment and collection of outcome, baseline, and other trial data, including any related processes to promote data quality (eg, duplicate measurements, training of assessors) and a description of study instruments (eg, questionnaires, laboratory tests) along with their reliability and validity, if known. Reference to where data collection forms can be found, if not in the protocol

For peer review only - http://bmjopen.bmj.com/site/about/guidelines.xhtml

NA		18b	Plans to promote participant retention and complete follow-up, including list of any outcome data to be collected for participants who discontinue or deviate from intervention protocols
NA	Data management	19	Plans for data entry, coding, security, and storage, including any related processes to promote data quality (eg, double data entry; range checks for data values). Reference to where details of data management procedures can be found, if not in the protocol
Y	Statistical methods	20a	Statistical methods for analysing primary and secondary outcomes. Reference to where other details of the statistical analysis plan can be found, if not in the protocol
NA		20b	Methods for any additional analyses (eg, subgroup and adjusted analyses)
NA		20c	Definition of analysis population relating to protocol non-adherence (eg, as randomised analysis), and any statistical methods to handle missing data (eg, multiple imputation)
	**Methods: Monitoring**		
NA	Data monitoring	21a	Composition of data monitoring committee (DMC); summary of its role and reporting structure; statement of whether it is independent from the sponsor and competing interests; and reference to where further details about its charter can be found, if not in the protocol. Alternatively, an explanation of why a DMC is not needed
NA		21b	Description of any interim analyses and stopping guidelines, including who will have access to these interim results and make the final decision to terminate the trial
NA	Harms	22	Plans for collecting, assessing, reporting, and managing solicited and spontaneously reported adverse events and other unintended effects of trial interventions or trial conduct
NA	Auditing	23	Frequency and procedures for auditing trial conduct, if any, and whether the process will be independent from investigators and the sponsor
	**Ethics and dissemination**		

Y	Research ethics approval	24	Plans for seeking research ethics committee/institutional review board (REC/IRB) approval
NA	Protocol amendments	25	Plans for communicating important protocol modifications (eg, changes to eligibility criteria, outcomes, analyses) to relevant parties (eg, investigators, REC/IRBs, trial participants, trial registries, journals, regulators)
Y	Consent or assent	26a	Who will obtain informed consent or assent from potential trial participants or authorised surrogates, and how (see Item 32)
NA		26b	Additional consent provisions for collection and use of participant data and biological specimens in ancillary studies, if applicable
NA	Confidentiality	27	How personal information about potential and enrolled participants will be collected, shared, and maintained in order to protect confidentiality before, during, and after the trial
Y	Declaration of interests	28	Financial and other competing interests for principal investigators for the overall trial and each study site
NA	Access to data	29	Statement of who will have access to the final trial dataset, and disclosure of contractual agreements that limit such access for investigators
NA	Ancillary and post-trial care	30	Provisions, if any, for ancillary and post-trial care, and for compensation to those who suffer harm from trial participation
Y	Dissemination policy	31a	Plans for investigators and sponsor to communicate trial results to participants, healthcare professionals, the public, and other relevant groups (eg, via publication, reporting in results databases, or other data sharing arrangements), including any publication restrictions
Y		31b	Authorship eligibility guidelines and any intended use of professional writers
NA		31c	Plans, if any, for granting public access to the full protocol, participant-level dataset, and statistical code
	**Appendices**		

For peer review only - http://bmjopen.bmj.com/site/about/guidelines.xhtml

NA	Informed consent materials	32	Model consent form and other related documentation given to participants and authorised surrogates
NA	Biological specimens	33	Plans for collection, laboratory evaluation, and storage of biological specimens for genetic or molecular analysis in the current trial and for future use in ancillary studies, if applicable

*It is strongly recommended that this checklist be read in conjunction with the SPIRIT 2013 Explanation & Elaboration for important clarification on the items. Amendments to the protocol should be tracked and dated. The SPIRIT checklist is copyrighted by the SPIRIT Group under the Creative Commons "Attribution-NonCommercial-NoDerivs 3.0 Unported" license.

For peer review only

BMJ Open

Increasing the Reach of Evidence-Based Interventions for Weight Management and Diabetes Prevention Among Medicaid Patients: Study Protocol for a Pilot Sequential Multiple Assignment Randomized Trial.

Journal:	*BMJ Open*
Manuscript ID	bmjopen-2023-075157.R1
Article Type:	Protocol
Date Submitted by the Author:	08-Aug-2023
Complete List of Authors:	Schlechter, Chelsey; University of Utah Health, Population Health Sciences; University of Utah Health Huntsman Cancer Institute<br>Del Fiol, Guilherme; University of Utah, Department of Biomedical Informatics<br>Jones, Dusti; University of Utah Health, Department of Population Health Sciences; University of Utah Health Huntsman Cancer Institute<br>Orleans, Brian; University of Utah Health Huntsman Cancer Institute<br>Gibson, Bryan; University of Utah, Department of Biomedical Informatics<br>Nahum-Shani, Inbal; University of Michigan<br>Maxfield, Ellen; University of Utah Health Care, Osher Center for Integrative Health; University of Utah Health, Department of Family & Preventative Medicine<br>Locke, Amy; University of Utah Health, Osher Center for Integrative Health; University of Utah Health, Department of Family & Preventive Medicine<br>Cornia, Ryan; University of Utah, Department of Biomedical Informatics<br>Bradshaw, Richard; University of Utah, Department of Biomedical Informatics<br>Wirth, Jennifer; University of Utah Health Huntsman Cancer Institute<br>Jaggers, Shanna; University of Utah Health Huntsman Cancer Institute<br>Lam, Cho; University of Utah Health, Department of Population Health Sciences; University of Utah Health Huntsman Cancer Institute<br>Wetter, David; University of Utah Health, Department of Population Health Sciences; University of Utah Health Huntsman Cancer Institute
**Primary Subject Heading**:	Public health
Secondary Subject Heading:	Evidence based practice, Diabetes and endocrinology
Keywords:	PUBLIC HEALTH, Obesity, Health Equity, Health informatics < BIOTECHNOLOGY & BIOINFORMATICS, Telemedicine < BIOTECHNOLOGY & BIOINFORMATICS

Authors: Chelsey R. Schlechter,[1,2+] Guilherme Del Fiol,[3] Dusti R. Jones,[1,2] Brian Orleans,[2] Bryan Gibson,[3] Inbal Nahum-Shani,[4] Ellen Maxfield,[5,6] Amy Locke,[5,6] Ryan Cornia,[3] Richard Bradshaw,[3] Jennifer Wirth,[2] Shanna J. Jaggers,[2] Cho Y. Lam,[1,2] David W. Wetter[1,2]

[1]Department of Population Health Sciences, University of Utah

[2]Huntsman Cancer Institute, University of Utah

[3]Department of Biomedical Informatics, University of Utah

[4]Institute for Social Research, University of Michigan

[5]Department of Family & Preventative Medicine, University of Utah

[6]Osher Center for Integrative Health, University of Utah

[+] Corresponding Author

Corresponding author's email address: Chelsey.schlechter@hci.utah.edu

Title: Increasing the Reach of Evidence-Based Interventions for Weight Management and Diabetes Prevention Among Medicaid Patients: Study Protocol for a Pilot Sequential Multiple Assignment Randomized Trial.

Abstract

Introduction: Over 40% of United States adults meet criteria for obesity, a major risk factor for cancer, cardiovascular disease, and diabetes. Obesity plays a critical role in health inequities, and disproportionately impacts populations that have been historically marginalized (e.g., low socioeconomic status, rural, some racial/ethnic minority groups). Evidence-based interventions (EBIs) for weight management exist but reach less than 3% of eligible individuals. The aims of this pilot randomized controlled trial are to evaluate feasibility and acceptability of dissemination strategies designed to increase reach of EBIs for weight management.

Methods and analysis: This study is a two-phase, Sequential Multiple Assignment Randomized Trial (SMART), conducted with 200 Medicaid patients. In Phase 1, patients will be individually randomized to single text message (TM1) or multiple text messages (TM+). Phase 2 is based on prior treatment

For peer review only

response. Patients who enroll in the EBI within 12 weeks of exposure to Phase 1 (i.e., responders) receive no further interventions. Patients in TM1 who do not enroll in the EBI within 12 weeks of exposure (i.e., TM1 non-responders) will be randomized to either TM1-Continued (i.e., no further TM) or TM&MAPS (i.e., no further TM, up to 2 Motivation And Problem Solving [MAPS] navigation calls) over the next 12 weeks. Patients in TM+ who do not enroll in the EBI (i.e., TM+ non-responders) will be randomized to either TM+Continued (i.e., monthly text messages) or TM+&MAPS (i.e., monthly text messages, plus up to 2 MAPS calls) over the next 12 weeks. Descriptive statistics will be used to characterize feasibility (e.g., proportion of patients eligible, contacted, and enrolled in the trial) and acceptability (e.g., participant opt-out, participant engagement with dissemination strategies, EBI Reach [i.e., the proportion of participants who enroll in EBI], adherence, effectiveness).

Ethics and dissemination: Study protocol was approved by the University of Utah Institutional Review Board (#00139694).

Strengths and limitations of this study

- Uses an innovative SMART design to optimize adaptive interventions.
- Potential reach and sustainability are enhanced by using dissemination strategy modalities (i.e., cellphone-based voice and text) that are ubiquitous among adults in the United States, including among historically marginalized populations, and are already utilized by payers and healthcare systems.
- Participant population was drawn from a single healthcare system in a single state.

Introduction

Approximately 40% of U.S. adults meet criteria for obesity (BMI ≥30),(1) and this proportion is expected to rise to nearly 50% in the next decade.(2) Obesity is a leading risk factor for multiple chronic diseases, including at least 13 different cancers,(3, 4, 5) Type 2 diabetes,(6, 7) cardiovascular disease,(8, 9) and premature death.(10) Additionally, obesity plays a critical role in health inequities, disproportionately impacting low socioeconomic status (SES), rural, and many racial/ethnic minority

For peer review only

populations.(11, 12, 13, 14, 15) For example, individuals with lower levels of education have higher obesity rates compared to those with higher levels of education.(16) Recent studies have found obesity rates of 41% among rural U.S. adults versus 32% among urban residents, and that rising BMI among rural populations has been a principal contributor to the obesity epidemic(17, 18) over the last 30 years. Finally, obesity is more prevalent among Latino adults compared to non-Latino White adults (45.6% and 41.4%, respectively).(15)

Evidence-based weight management and diabetes prevention programs. Recommendations from the United States Preventive Services Task Force and others highlight that adults with obesity should engage in comprehensive, multicomponent behavioral interventions addressing weight, diet, and physical activity.(19, 20, 21, 22) Components of these behavioral evidence-based interventions (EBIs) include improving diet and nutrition, increasing physical activity, and behavioral strategies such as goal setting and self-monitoring.(23) The Diabetes Prevention Program (DPP) is among the most well-known EBIs for weight management, with substantial evidence for weight loss, increased physical activity, decreased daily energy intake, and decreased incidence of Type 2 diabetes.(24) The DPP can be delivered via in-person or digital modalities, and there are currently over 1800 Centers for Disease Control and Prevention (CDC) recognized DPP programs across the U.S.(25) Despite their effectiveness, DPPs are grossly underutilized,(26) reaching only 3% of eligible individuals.(27) Furthermore, there are substantial disparities in DPP access (14.6% of rural counties contain a DPP site vs. 48.4% for urban counties)(28) and engagement among historically marginalized populations (e.g., low SES, Latino).(26, 29) Barriers to participation for in-person delivered DPPs among underserved populations include the need to travel long distances, and the lack of transportation, financial resources, childcare, and time. Fortunately, digital DPP delivery modalities such as the telephone, internet, and smartphones have yielded weight loss and other outcomes comparable to in-person DPPs, with greater engagement and lower attrition in digital programs.(24, 26, 30, 31, 32, 33, 34) Consistent with those findings, the Community Preventive Services Task Force concluded that "Technology-Supported Multicomponent Coaching or Counseling Interventions" are recommended for both weight loss and weight loss maintenance,(20) and that

For peer review only

technology-supported programs may increase access among populations that have transportation issues, unusual work schedules, or are rural. Similarly, the National Academy of Medicine report on obesity treatment identified key research recommendations that included improving access, employing technology to increase reach, and reducing health disparities.(21)

Dissemination Strategies to Increase EBI Reach. Dissemination strategies are designed to increase the reach of EBIs by increasing individual motivation and capability to access and engage with the interventions.(35, 36) Many strategies to increase the reach of weight management EBIs rely on dissemination strategies at the point of care (e.g., provider referral to EBI).(37, 38, 39) However, other potent strategies for increasing reach include proactive, population health management approaches that can remove the burden from clinical staff to offer the referral, and can also reach out to all appropriate patients without the need for a clinical visit to trigger the referral offer. Population health management approaches have increased referrals to EBIs for weight management(27, 37) and enrollment among eligible participants.(40, 41)

Digital and telehealth dissemination strategies such as text messaging and phone-based health coaching are promising approaches to improve dissemination of EBIs for weight management, and have near ubiquitous reach given 97% of Americans own a cell phone, including 97% of individuals with income less than $30,000 per year, 94% of rural individuals, and nearly 100% of Hispanic individuals.(42) Text messaging has been effective for improving numerous health behaviors (e.g., physical activity, nutrition, and tobacco cessation), and for improving attendance and compliance with behavioral interventions.(43, 44, 45, 46, 47, 48) Similarly, personalized support to address barriers to care via patient navigation has been shown to improve completion rates for cancer screening, increase adherence to medication, improve compliance to treatment and follow-up visits, improve biomarkers for diabetes, and increase enrollment in EBIs.(49, 50, 51, 52) However, there is limited research on the effectiveness of these approaches for increasing reach of EBIs for weight management among historically marginalized populations.

For peer review only

Adaptive interventions. Adaptive interventions contain a sequence of individually tailored decision rules that specify the intensity or type of intervention at critical points in the delivery of care.(53, 54, 55). Modifying intensity or type of intervention over time can improve outcomes if an individual is not responding, or may reduce costs and burden if intensive treatment is not necessary.(56) For example, a stepped care model (a type of adaptive intervention) can conserve scarce resources and reduce burden on patients. Sequential Multiple Assignment Randomized Trials (SMART(57, 58)) are a type of experimental design used to inform the dosage, type, delivery, and timing of adaptive interventions.

Aims and objectives. The overall aims of this pilot study are to evaluate the feasibility and acceptability of the dissemination strategies and study procedures. Feasibility will be measured by the proportion of people who were eligible, contacted, and randomized in the trial. Acceptability will be assessed by rate of participant opt-out, participant engagement with dissemination strategies, EBI Reach by randomization condition, and EBI adherence and effectiveness among participants who enroll in the EBI.

Methods and Analysis

The protocol for this pilot study has been reviewed and approved by the University of Utah Institutional Review Board (IRB). Data collection was planned to begin March 2022 and end in May 2023. The trial is registered at clinicaltrials.gov (NCT05666323). This manuscript adheres to the Standards Protocol Items: Recommendations for Interventional Trials (SPIRIT).

Settings and Participants.

Participants will be 200 adults 1) age ≥ 18, 2) BMI ≥ 30; 2) have had a primary care appointment within University of Utah Health (UHealth) within the past 12 months; 3) speak English or Spanish as their primary language; 4) have a current Utah address; and 5) are insured through Medicaid by the University of Utah Health Plans. Exclusion criteria include 1) a diagnosis of Type 1 or Type 2 diabetes, 2) documented current pregnancy, and 3) no phone number listed in the EHR.

Study Design.

For peer review only

This trial is a two-phase, Sequential Multiple Assignment Randomized Trial (SMART) with 200 Medicaid patients from a single academic medical system (Figure 1). In the first phase, participants will be individually randomized to receive a single text message (TM1), or multiple text messages (TM+) offering free enrollment in a digital EBI for weight management.

Phase 2 dissemination strategies are randomized based on prior dissemination strategy response. Participants in TM1 who do not opt out or enroll in EBI by 12 weeks will be randomized to either TM1-Continued (no further TM) or TM1 & MAPS (no further TM and up to 2 Motivation And Problem Solving [MAPS] calls from a patient navigator). Participants in TM+ who do not opt out or enroll in the EBI by 12 weeks will be randomized to either TM+ Continued (monthly text messages for the next 12 weeks) or TM+ & MAPS (monthly text messages for the next 12 weeks, plus up to 2 MAPS calls from a patient navigator). Descriptions of participant interventions are listed in Table 1.

Table 1: Randomization Condition Description

	**Randomization Condition**	**Description**
**Phase 1**		
	Single text message (TM1)	Participants receive a single text message that includes a motivational message, the EBI phone number, and a two-touch response that directly connects interested participants to EBI enrollment.
	Multiple text message (TM+)	Participants receive messages once per week for the first 2 weeks, then 1 message every 3 weeks for the remaining 10 weeks (up to 5 texts total). The text messages include a motivational message, the EBI phone number, and a simple two-touch response that directly connects interested participants to EBI enrollment.
**Phase 2**		
For TM1 non-responding participants only.	Single text message continued (TM1-Cont)	Participants receive no additional text messages.
	Single text message & Motivation And Problem Solving (MAPS) (TM1 & MAPS)	Participants receive no additional text messages and receive up to 2 MAPS counselling calls from a health coach.
For TM+ non-responding participants only.	Multiple text message continued (TM+-Cont)	Participants receive text messages every 4 weeks for the next 12 weeks (up to 3 text messages total).
	Multiple text message & MAPS (TM+ & MAPS)	Participants receive text messages every 4 weeks for the next 12 weeks (up to 3 text messages total)

	plus up to 2 MAPS counselling calls from a health coach.

Dissemination Strategies

Text Messaging. Participants will receive bidirectional Health Insurance Portability and Accountability Act (HIPAA) compliant text messages that include a brief motivational message, the opportunity to enroll in the EBI, and the opportunity to opt-out of receiving further dissemination strategies.

Motivation And Problem Solving (MAPS) Health Coaching. Participants will receive up to two brief calls from health coaches trained in using MAPS, a coaching/counselling approach designed to help participants address the larger context in which behavior change occurs, identify and address barriers to change, and motivate participants towards a long-term goal of change. MAPS is a holistic, dynamic behavioral approach that integrates two empirically validated approaches (motivational interviewing(59, 60) and problem solving/social cognitive strategies(61, 62, 63)) into a coordinated intervention with demonstrated efficacy for addressing the entire process of considering behavior change, attempting to change, and maintaining change.(50, 63, 64, 65, 66) MAPS employs a "wellness" approach that in addition to focusing on the target behavior, provides practical advice and connections to additional services whenever possible for life events, stressors, and concerns that impact the lives of rural and other historically marginalized individuals (e.g., isolation, stress, depression, financial barriers, etc.,). MAPS is based on substantial evidence that motivation fluctuates frequently and rapidly, and as such, is designed to fluidly respond to those changes.(67, 68, 69, 70) MAPS health coaching calls will be delivered by two bilingual health coaches on the research team with at least 20 hours of training in MAPS delivery.

Digital EBI for Diabetes Prevention and Weight Management.

Participants who enroll in the EBI will receive either a digital DPP or digital weight management program based on criteria for meeting prediabetes. Participants will be evaluated for risk of prediabetes using the American Diabetes Association (ADA) and CDC Prediabetes Risk Test(71) at the time of

For peer review only - http://bmjopen.bmj.com/site/about/guidelines.xhtml

enrolling in the EBI. The test uses only self-report health trait data (e.g., family history, height, weight, physical activity level, health history of hypertension and gestational diabetes) and does not require testing data (i.e., hemoglobin A1C or cholesterol levels). Participants who meet criteria for prediabetes based on the risk test will be triaged into a digital DPP program; participants who do not meet criteria will be triaged to a digital weight management program. Both programs will be delivered by EBI vendor, IncentaHEALTH. Based on national data on eligibility for the DPP among individuals with obesity and data from the EBI provider, we estimate that ~50% of participants will be triaged to DPP and ~50% to weight management.(72)

The digital DPP offered to study participants is a CDC fully recognized program. The digital weight management program, for all participants who do not meet criteria for the DPP, uses the same general components as the DPP. Both programs are designed to increase intake of fruits, vegetables, lean protein and complex carbohydrates while monitoring portion size. Physical activity recommendations include cardiovascular and strength training, with a goal of approximately 30 minutes 5-6 days/week. Both programs are offered in English and Spanish, based on Social Cognitive Theory, and provide personalized health coaching. Both include a website, a Bluetooth wireless scale for weight monitoring, daily email/text message support, easy to follow video exercise plans, customized meal plans, grocery lists, incorporate skill building, and incorporate social accountability. Participants have unlimited access to Health Coaches, who have at least a Bachelor's degree, along with certifications, in personal training, nutrition, or wellness.

Recruitment.

The flow of participants through the study is described in Figure 2. Participants who meet inclusion criteria will be identified from the University of Utah Health (UHealth) Electronic Data Warehouse (EDW). Participant demographic, preferred language, and relevant health data, as well as participant contact information (i.e., phone number, address, email address) will be extracted from the EDW for all eligible participants. Participants who meet exclusionary criteria will be removed from the eligible population. We will use quota sampling from the eligible population to achieve a stratified

For peer review only

random sample aimed to offer balance in the marginal distributions of key participant demographic characteristics. These margins include 1) male/female sex (1:1 ratio), 2) urban/rural according to participant zip code and the 2010 Rural Urban Commuting Area (RUCA) codes (>= 4 defined as rural) (1:1 ratio), and 3) Latino with English as preferred language, Latino with Spanish as preferred language, and non-Latino with English as preferred language (1:1:3 ratio).

Sampled patients randomized to TM1 or TM+ will be sent an e-mail informing them that they will be receiving communications from UHealth about the availability of a digital EBI for weight management. The e-mail will provide patients with information about how they can opt-out if they do not wish to receive these communications. Patients who do not have an email in the EHR will be sent the opt-out information via mail. Patients who opt-out of the study receive no further interventions.

Randomization and Blinding.

In Phase 1 (Figure 1), participants will be randomly assigned to TM1 or TM+ in a 1:1 ratio. In Phase 2 (Figure 1), participants who opt out or who enroll in the EBI will receive no further dissemination strategies and will not be re-randomized. Participants in TM1 did not opt-out and who did not enroll in the EBI will be randomized to TM1-Cont or TM1 & MAPS at a ratio of 1:1. Participants in TM+ who did not opt-out and did not enroll in the EBI will be randomized to TM+-Cont or TM+ & MAPS at a ratio of 1:1. All randomization sequences will be generated via SAS 9.4 to consist of random permuted blocks with random block sizes, thus ensuring balance in the allocation over time.

Data Collection and Outcomes.

Participant demographic and baseline health data (e.g., age, gender, BMI, pregnancy status, medical codes for Type 1 and Type 2 diabetes, zip code, preferred language, race/ethnicity) will be obtained from the UHealth EDW.

Feasibility outcomes. Feasibility will be assessed using the proportion of participants who were eligible, contacted, and enrolled in the trial.

Acceptability outcomes. Acceptability will be assessed using participant opt-out, participant engagement with dissemination strategies, EBI reach, and EBI effectiveness. Participant opt-out is

For peer review only - http://bmjopen.bmj.com/site/about/guidelines.xhtml

assessed by the absolute number and proportion of eligible participants who opt-out during Phase 1 and Phase 2. Participant engagement will be assessed as 1) the number and proportion of participants who respond to text messages with a response other than opt-out, 2) the number and proportion of eligible participants who talk to a MAPS health coach, 3) the number and proportion of eligible participants accept connection to the EBI, and 4) the number and proportion of participants who talk to the EBI personnel. EBI reach is assessed by the number and proportion of eligible participants who officially enroll in the EBI. Enrollment is defined in accordance with the CDC definition for recognized virtual programs, which consists of the participant setting a password for the program. Enrollment will be obtained from the EBI vendor (IncentaHEALTH). EBI adherence and effectiveness are measured by participant adherence to the EBI components, percentage of body-weight lost, and change in BMI, and will be collected for 12 weeks following EBI enrollment. Adherence and effectiveness data will be obtained from the EBI vendor (IncentaHEALTH)

Patient and Public Involvement.

This study was developed and implemented in collaboration with the University of Utah Wellness and Integrative Health Program, which supports population health initiatives through integration of wellness services into patient care and health plan offerings. The program currently manages lifestyle behavior change programs (including the Diabetes Prevention Program) for UHealth patients and University of Utah faculty, staff, and students. The dissemination strategies, the feasibility and acceptability outcomes, and study procedures were developed in collaboration with the University of Utah Wellness and Integrative Health Program and investigators at the University of Utah. Patients were not directly involved in the development of this pilot study.

Ethics and dissemination.

All procedures performed in studies involving human participants will be conducted in accordance with the ethical standards of the institutional and/or national research committee and with the 1964 Helsinki declaration and its later amendments or comparable ethical standards. The protocol for this study was approved by the University of Utah Institutional Review Board (00139694). Materials used to

For peer review only

conduct the study are not currently publicly available. Materials may be requested by emailing the corresponding author. Study results will be dissemination via peer reviewed publications and manuscripts, as well as to the health system and community partners via lay reports and presentations.

Data Analysis Plan.

Our primary study aim is to evaluate the acceptability and feasibility of dissemination strategies to increase of EBIs for weight management.

Primary Analyses Phase 1. The analysis of acceptability and feasibility at 12 weeks will include all participants enrolled in the study. We will use descriptive statistics (mean, standard deviation (SD), confidence interval) to evaluate each indicator of feasibility and acceptability by dissemination strategies (i.e., TM1, TM+).

Primary Analyses Phase 2. The analysis of feasibility and acceptability at 24-weeks will be conducted using only data from those participants who did not opt out or enroll in the EBIs by 12 weeks (i.e., non-responders). We will use descriptive statistics to evaluate each indicator of feasibility and acceptability by dissemination strategies (i.e., TM1-Cont, TM1 & MAPS, TM+-Cont, TM+ & MAPS).

Power Analysis.

The sample size (N = 200) for the current project was determined based on feasibility of sample size recruitment from a single Medicaid provider and completing project procedures in the study timeframe. As such, power calculations were not conducted for this pilot trial.

Strengths and Limitations.

The proposed pilot study uses an innovative SMART design to optimize adaptive interventions to increase reach of already existing EBIs for weight management among historically marginalized populations. Potential reach and sustainability are enhanced by using dissemination strategy modalities (i.e., cellphone-based voice and text) that are ubiquitous among adults in the United States, including among historically marginalized populations, and are already utilized by payers and healthcare systems. The participant population was drawn from a single healthcare system in a single state which may limit

For peer review only

generalizability of results. However, the proposed study population will represent a diverse sample based on sex, ethnicity, primary language, and rurality.

Conclusion

The proposed pilot project is a two-phase SMART with the objective of evaluating acceptability and feasibility of dissemination strategies designed to increase the reach of digital EBIs for weight management among Medicaid patients. Accomplishing the aims of the proposed study will provide critical data regarding the feasibility and acceptability of pragmatic and scalable dissemination strategies designed to increase the uptake of digital EBIs for obesity among historically marginalized populations, and inform the conduct of a future, fully powered randomized trial.

Registration details: The trial is registered at clinicaltrials.gov (NCT05666323).

Funding statement. This work was supported through the Ben B. and Iris M. Margolis Foundation, the National Center for Advancing Translational Sciences of the National Institutes of Health (UL1TR002538), the National Cancer Institute (P30CA042014), and the Huntsman Cancer Foundation.

Competing interests. The authors declare no competing interests.

Author contribution. DWW acquired financial support for the project. CRS, GDF, BO, BG, EM, AL, JW, CYL, RC, RB, SJ, IS, and DWW contributed to study conceptualization. All authors contributed to the development or design of the methodology, dissemination strategies, study procedures, study outcomes, or analytic plan. CRS and DRJ drafted the original manuscript. All authors reviewed, edited, and approved the final manuscript.

Figure caption.

Figure 1: Sequential multiple assignment randomized trial (SMART) study design

Figure 2: Patient flow diagram

References

For peer review only

1. Hales CM, Carroll MD, Fryar CD, Ogden CL. Prevalence of Obesity and Severe Obesity Among Adults: United States, 2017–2018. NCHS Data Brief. 2020;no. 360(1-7).
2. Ward ZJ, Bleich SN, Cradock AL, Barrett JL, Giles CM, Flax C, et al. Projected U.S. State-Level Prevalence of Adult Obesity and Severe Obesity. N Engl J Med. 2019;381(25):2440-50.
3. Islami F, Goding Sauer A, Miller KD, Siegel RL, Fedewa SA, Jacobs EJ, et al. Proportion and number of cancer cases and deaths attributable to potentially modifiable risk factors in the United States. CA: A Cancer Journal for Clinicians. 2018;68(1):31-54.
4. Islami F, Goding Sauer A, Gapstur SM, Jemal A. Proportion of Cancer Cases Attributable to Excess Body Weight by US State, 2011-2015. JAMA Oncology. 2019;5(3):384-92.
5. McTiernan A, Friedenreich CM, Katzmarzyk PT, Powell KE, Macko R, Buchner D, et al. Physical Activity in Cancer Prevention and Survival: A Systematic Review. Med Sci Sports Exerc. 2019;51(6):1252-61.
6. Verma S, Hussain ME. Obesity and diabetes: An update. Diabetes Metab Syndr. 2017;11(1):73-9.
7. Mokdad AH, Ford ES, Bowman BA, Dietz WH, Vinicor F, Bales VS, et al. Prevalence of obesity, diabetes, and obesity-related health risk factors, 2001. Jama. 2003;289(1):76-9.
8. Powell-Wiley TM, Poirier P, Burke LE, Després J-P, Gordon-Larsen P, Lavie CJ, et al. Obesity and cardiovascular disease: a scientific statement from the American Heart Association. Circulation. 2021;143(21):e984-e1010.
9. Khan SS, Ning H, Wilkins JT, Allen N, Carnethon M, Berry JD, et al. Association of Body Mass Index With Lifetime Risk of Cardiovascular Disease and Compression of Morbidity. JAMA Cardiol. 2018;3(4):280-7.
10. Hruby A, Manson JE, Qi L, Malik VS, Rimm EB, Sun Q, et al. Determinants and Consequences of Obesity. Am J Public Health. 2016;106(9):1656-62.
11. AHRQ. 2018 National Healthcare Quality and Disparities. Rockville, MD: Agency for Healthcare Research and Quality; September 2019. Contract No.: AHRQ Publication No. 19-0070-EF.
12. Clarke AR, Goddu AP, Nocon RS, Stock NW, Chyr LC, Akuoko JAS, et al. Thirty years of disparities intervention research: what are we doing to close racial and ethnic gaps in health care? Med Care. 2013;51(11):1020-6.
13. Crosby RA, Wendel ML, Vanderpool RC, Casey BR, editors. Rural populations and health: determinants, disparities, and solutions. San Francisco, CA: Jossey-Bass; 2012.
14. National Center for Health Statistics. Health, United States, 2011: With Special Feature on Socioeconomic Status and Health. Hyattsville, MD: National Center for Health Statistics (US); 2012.
15. Akinbami LJ, Chen TC, Davy O, Ogden CL, Fink S, Clark J, et al. National Health and Nutrition Examination Survey, 2017-March 2020 Prepandemic File: Sample Design, Estimation, and Analytic Guidelines. Vital Health Stat 1. 2022(190):1-36.
16. Stierman B, Afful J, Carroll MD, Chen T-C, Davy O, Fink S, et al. National Health and Nutrition Examination Survey 2017–March 2020 prepandemic data files development of files and prevalence estimates for selected health outcomes. 2021.
17. NCD Risk Factor Collaboration. Rising rural body-mass index is the main driver of the global obesity epidemic in adults. Nature. 2019;569(7755):260.

For peer review only

18. Wen M, Fan JX, Kowaleski-Jones L, Wan N. Rural–urban disparities in obesity prevalence among working age adults in the United States: exploring the mechanisms. American Journal of Health Promotion. 2018;32(2):400-8.
19. Jensen MD, Ryan DH, Apovian CM, Ard J, Comuzzie AG, Donato KA, et al. 2013 AHA/ACC/TOS guideline for the management of overweight and obesity in adults: a report of the American College of Cardiology/American Heart Association task force on practice guidelines and the obesity society. Circulation. 2014;129(25 Suppl 2):S102-S38.
20. Community Preventive Services Task Force. Obesity: Technology-Supported Multicomponent Coaching or Counseling Interventions to Maintain Weight Loss. Retrieved from https://www.thecommunityguide.org/findings/obesity-technology-supported-multicomponent-coaching-or-counseling-interventions-maintain.
21. Heymsfield S, Aronne LJ, Eneli I, Kumar RB, Michalsky M, Walker E, et al. Clinical Perspectives on Obesity Treatment: Challenges, Gaps, and Promising Opportunities. NAM Perspectives. 2018.
22. LeBlanc ES, Patnode CD, Webber EM, Redmond N, Rushkin M, O'Connor EA. Behavioral and pharmacotherapy weight loss interventions to prevent obesity-related morbidity and mortality in adults: Updated evidence report and systematic review for the US Preventive Services Task Force. Jama. 2018;320(11):1172-91.
23. Curry SJ, Krist AH, Owens DK, Barry MJ, Caughey AB, Davidson KW, et al. Behavioral weight loss interventions to prevent obesity-related morbidity and mortality in adults: US preventive services task force recommendation statement. Jama. 2018;320(11):1163-71.
24. Dunkley AJ, Bodicoat DH, Greaves CJ, Russell C, Yates T, Davies MJ, et al. Diabetes prevention in the real world: effectiveness of pragmatic lifestyle interventions for the prevention of type 2 diabetes and of the impact of adherence to guideline recommendations: a systematic review and meta-analysis. Diabetes care. 2014;37(4):922-33.
25. CDC. Diabetes Prevention Recognition Program – Registry of Recognized Organizations [Available from: https://nccd.cdc.gov/DDT_DPRP/Registry.aspx.
26. Ali MK, Bullard KM, Imperatore G, Benoit SR, Rolka DB, Albright AL, et al. Reach and use of diabetes prevention services in the United States, 2016-2017. JAMA network open. 2019;2(5):e193160-e.
27. Alva ML, Chakkalakal RJ, Moin T, Galaviz KI. The Diabetes Prevention Gap And Opportunities To Increase Participation In Effective Interventions. Health Aff (Millwood). 2022;41(7):971-9.
28. Ariel-Donges AH, Gordon EL, Dixon BN, Eastman AJ, Bauman V, Ross KM, et al. Rural/urban disparities in access to the National Diabetes Prevention Program. Translational behavioral medicine. 2019.
29. Ritchie ND, Christoe-Frazier L, McFann KK, Havranek EP, Pereira RI. Effect of the National Diabetes Prevention Program on Weight Loss for English- and Spanish-Speaking Latinos. American Journal of Health Promotion. 2017;32(3):812-5.
30. Moin T, Damschroder LJ, AuYoung M, Maciejewski ML, Havens K, Ertl K, et al. Results From a Trial of an Online Diabetes Prevention Program Intervention. American Journal of Preventive Medicine. 2018;55(5):583-91.

For peer review only

31. Mudaliar U, Zabetian A, Goodman M, Echouffo-Tcheugui JB, Albright AL, Gregg EW, et al. Cardiometabolic risk factor changes observed in diabetes prevention programs in US settings: a systematic review and meta-analysis. PLoS medicine. 2016;13(7).
32. Taetzsch A, Gilhooly CH, Bukhari A, Das SK, Martin E, Hatch AM, et al. Development of a Videoconference-Adapted Version of the Community Diabetes Prevention Program, and Comparison of Weight Loss With In-Person Program Delivery. Military Medicine. 2019;184(11-12):647-52.
33. Tice J, Chapman R, Shore KK, Seidner M, Ollendorf D, Weissberg J, et al. Diabetes Prevention Programs: effectiveness and value: final evidence report and meeting summary. Institute for Clinical and Economic Review; 2018.
34. Kim SE, Sweet CMC, Gibson E, Madero EN, Rubino B, Morrison J, et al. Evaluation of a digital diabetes prevention program adapted for the Medicaid population: study design and methods for a non-randomized, controlled trial. Contemporary clinical trials communications. 2018;10:161-8.
35. Lomas J. Diffusion, dissemination, and implementation: who should do what? Ann N Y Acad Sci. 1993;703:226-35; discussion 35-7.
36. McCormack L, Sheridan S, Lewis M, Boudewyns V, Melvin CL, Kistler C, et al. Communication and dissemination strategies to facilitate the use of health-related evidence. Evid Rep Technol Assess (Full Rep). 2013(213):1-520.
37. Holliday CS, Williams J, Salcedo V, Kandula NR. Clinical Identification and Referral of Adults With Prediabetes to a Diabetes Prevention Program. Prev Chronic Dis. 2019;16:E82.
38. Nhim K, Khan T, Gruss S, Wozniak G, Kirley K, Schumacher P, et al. Facilitators to referrals to CDC's National Diabetes Prevention Program in primary care practices and pharmacies: DocStyles 2016-2017. Prev Med. 2021;149:106614.
39. Kirley K, Khan T, Aquino G, Brown A, Meier S, Chambers N, et al. Using a certified electronic health record technology platform to screen, test and refer patients with prediabetes. JAMIA Open. 2021;4(4):ooab101.
40. Porter G, Michaud TL, Schwab RJ, Hill JL, Estabrooks PA. Reach Outcomes and Costs of Different Physician Referral Strategies for a Weight Management Program Among Rural Primary Care Patients: Type 3 Hybrid Effectiveness-Implementation Trial. JMIR Formative Research. 2021;5(10):e28622.
41. Wilson KE, Michaud TL, Almeida FA, Schwab RJ, Porter GC, Aquilina KH, et al. Using a population health management approach to enroll participants in a diabetes prevention trial: reach outcomes from the PREDICTS randomized clinical trial. Translational behavioral medicine. 2021;11(5):1066-77.
42. Center PR. Mobile Fact Sheet 2021 [Available from: https://www.pewresearch.org/internet/fact-sheet/mobile/.
43. Cole-Lewis H, Kershaw T. Text messaging as a tool for behavior change in disease prevention and management. Epidemiologic reviews. 2010:mxq004.
44. Gurol-Urganci I, de Jongh T, Vodopivec-Jamsek V, Atun R, Car J. Mobile phone messaging reminders for attendance at healthcare appointments. Cochrane Database of Systematic Reviews. 2013(12).
45. Head KJ, Noar SM, Iannarino NT, Harrington NG. Efficacy of text messaging-based interventions for health promotion: a meta-analysis. Social Science & Medicine. 2013;97:41-8.

For peer review only

46. Schwebel FJ, Larimer ME. Using text message reminders in health care services: A narrative literature review. Internet Interventions. 2018;13:82-104.
47. Vodopivec-Jamsek V, de Jongh T, Gurol-Urganci I, Atun R, Car J. Mobile phone messaging for preventive health care. The Cochrane Library. 2012.
48. USDHHS. Using Health Text Messages To Improve Consumer Health Knowledge, Behaviors, And Outcomes: An Environmental Scan. Rockville, MD: US Department of Health and Human Services; 2014.
49. McBrien KA, Ivers N, Barnieh L, Bailey JJ, Lorenzetti DL, Nicholas D, et al. Patient navigators for people with chronic disease: A systematic review. PloS one. 2018;13(2):e0191980-e.
50. Vinci C, Lam C, Schlechter CR, Shono Y, Vidrine JI, Wetter DW. Increasing treatment enrollment among smokers who are not motivated to quit: a randomized clinical trial. Translational Behavioral Medicine. 2021.
51. Peart A, Lewis V, Brown T, Russell G. Patient navigators facilitating access to primary care: a scoping review. BMJ open. 2018;8(3):e019252.
52. Valaitis RK, Carter N, Lam A, Nicholl J, Feather J, Cleghorn L. Implementation and maintenance of patient navigation programs linking primary care with community-based health and social services: a scoping literature review. BMC health services research. 2017;17(1):116.
53. Almirall D, Collins LM, Murphy SA. Introduction to adaptive interventions and SMART study design principles. COPTR Workshop on Adaptive Interventions 2011.
54. Bierman KL, Nix RL, Maples JJ, Murphy SA. Examining clinical judgment in an adaptive intervention design: The fast track program. Journal of Consulting and Clinical Psychology. 2006;74(3):468.
55. McKay JR. Is there a case for extended interventions for alcohol and drug use disorders? Addiction. 2005;100(11):1594-610.
56. Lei H, Nahum-Shani I, Lynch K, Oslin D, Murphy S. A" SMART" design for building individualized treatment sequences. Annual review of clinical psychology. 2012;8:21-48.
57. Lavori PW, Dawson R. Introduction to dynamic treatment strategies and sequential multiple assignment randomization. Clinical Trials. 2014:1740774514527651.
58. Murphy SA. An experimental design for the development of adaptive treatment strategies. Statistics in medicine. 2005;24(10):1455-81.
59. Miller WR, Rollnick S. Motivational Interviewing: Preparing People to Change Addictive Behavior. New York: The Guildford Press; 1991.
60. Miller WR, Rollnick S. Motivational Interviewing (2nd edition). New York: The Guilford Press; 2002.
61. Marlatt G, Donovan D, editors. Relapse prevention: Maintenance strategies in the treatment of addictive behaviors (2nd edition). New York: Guilford Press; 2005.
62. Witkiewitz K, Marlatt GA. Relapse prevention for alcohol and drug problems: That was Zen, this is Tao. American Psychologist. 2004;59(4):224-35.
63. Reitzel LR, Vidrine JI, Businelle MS, Kendzor DE, Costello TJ, Li Y, et al. Preventing postpartum smoking relapse among diverse low-income women: a randomized clinical trial. Nicotine Tob Res. 2010;12(4):326-35.

For peer review only

64. Correa-Fernández V, Díaz-Toro EC, Reitzel LR, Guo L, Chen M, Li Y, et al. Combined treatment for at-risk drinking and smoking cessation among Puerto Ricans: A randomized clinical trial. Addictive Behaviors. 2017;65:185-92.
65. McClure JB, Westbrook E, Curry SJ, Wetter DW. Proactive, motivationally enhanced smoking cessation counseling among women with elevated cervical cancer risk. Nicotine & Tobacco Research. 2005;7(6):881-9.
66. Wetter DW, Mazas C, Daza P, Nguyen L, Fouladi RT, Li Y, et al. Reaching and treating Spanish-speaking smokers through the National Cancer Institute's Cancer Information Service. A randomized controlled trial. Cancer. 2007;109(2 Suppl):406-13.
67. Conroy DE, Elavsky S, Hyde AL, Doerksen SE. The dynamic nature of physical activity intentions: a within-person perspective on intention-behavior coupling. Journal of Sport and Exercise Psychology. 2011;33(6):807-27.
68. Maher JP, Dunton GF. Within-day time-varying associations between motivation and movement-related behaviors in older adults. Psychology of Sport and Exercise. 2020;47:101522.
69. Vartanian LR, Pinkus RT, Smyth JM. Experiences of weight stigma in everyday life: Implications for health motivation. Stigma and Health. 2018;3(2):85-92.
70. Vidrine JI, Reitzel LR, Figueroa P, Velasquez MM, Mazas CA, Cinciripini PM, et al. Motivation And Problem Solving (MAPS): Motivationally-based skills training for treating substance use. Cogn Behav Pract. 2013;20(4):501-16.
71. Centers for Disease Control Prevention ADA. Predaibetes Risk Test [Available from: https://www.cdc.gov/diabetes/prevention/pdf/Prediabetes-Risk-Test-Final.pdf.
72. Bullard KM, Saydah SH, Imperatore G, Cowie CC, Gregg EW, Geiss LS, et al. Secular changes in US prediabetes prevalence defined by hemoglobin A1c and fasting plasma glucose: National Health and Nutrition Examination Surveys, 1999–2010. Diabetes Care. 2013;36(8):2286-93.

For peer review only

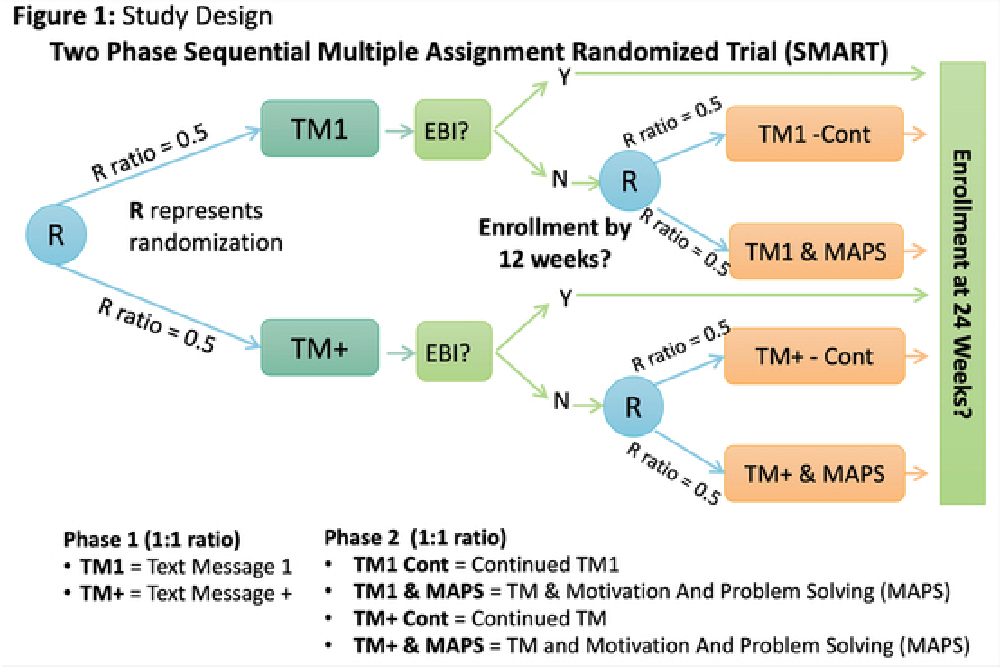

Figure 1: Study Design

195x130mm (72 x 72 DPI)

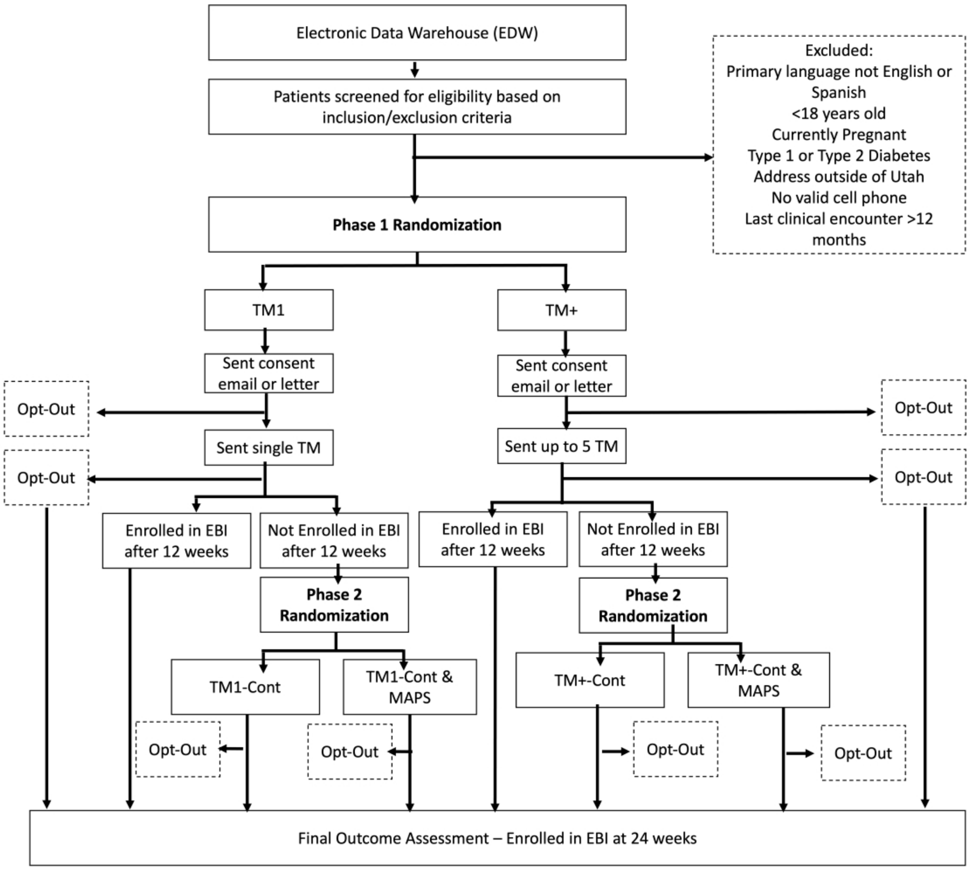

Figure 2: Consort Diagram

361x321mm (330 x 330 DPI)

SPIRIT
STANDARD PROTOCOL ITEMS: RECOMMENDATIONS FOR INTERVENTIONAL TRIALS

SPIRIT 2013 Checklist: Recommended items to address in a clinical trial protocol and related documents*

	Section/item	ItemNo	Description
	**Administrative information**		
Y	Title	1	Descriptive title identifying the study design, population, interventions, and, if applicable, trial acronym
Y	Trial registration	2a	Trial identifier and registry name. If not yet registered, name of intended registry
NA		2b	All items from the World Health Organization Trial Registration Data Set
NA	Protocol version	3	Date and version identifier
Y	Funding	4	Sources and types of financial, material, and other support
Y	Roles and responsibilities	5a	Names, affiliations, and roles of protocol contributors
Y		5b	Name and contact information for the trial sponsor
Y		5c	Role of study sponsor and funders, if any, in study design; collection, management, analysis, and interpretation of data; writing of the report; and the decision to submit the report for publication, including whether they will have ultimate authority over any of these activities
NA		5d	Composition, roles, and responsibilities of the coordinating centre, steering committee, endpoint adjudication committee, data management team, and other individuals or groups overseeing the trial, if applicable (see Item 21a for data monitoring committee)
	**Introduction**		
Y	Background and rationale	6a	Description of research question and justification for undertaking the trial, including summary of relevant studies (published and unpublished) examining benefits and harms for each intervention

For peer review only

Y		6b	Explanation for choice of comparators
Y	Objectives	7	Specific objectives or hypotheses
Y	Trial design	8	Description of trial design including type of trial (eg, parallel group, crossover, factorial, single group), allocation ratio, and framework (eg, superiority, equivalence, noninferiority, exploratory)
	**Methods: Participants, interventions, and outcomes**		
Y	Study setting	9	Description of study settings (eg, community clinic, academic hospital) and list of countries where data will be collected. Reference to where list of study sites can be obtained
Y	Eligibility criteria	10	Inclusion and exclusion criteria for participants. If applicable, eligibility criteria for study centres and individuals who will perform the interventions (eg, surgeons, psychotherapists)
Y	Interventions	11a	Interventions for each group with sufficient detail to allow replication, including how and when they will be administered
NA		11b	Criteria for discontinuing or modifying allocated interventions for a given trial participant (eg, drug dose change in response to harms, participant request, or improving/worsening disease)
NA		11c	Strategies to improve adherence to intervention protocols, and any procedures for monitoring adherence (eg, drug tablet return, laboratory tests)
NA		11d	Relevant concomitant care and interventions that are permitted or prohibited during the trial
Y	Outcomes	12	Primary, secondary, and other outcomes, including the specific measurement variable (eg, systolic blood pressure), analysis metric (eg, change from baseline, final value, time to event), method of aggregation (eg, median, proportion), and time point for each outcome. Explanation of the clinical relevance of chosen efficacy and harm outcomes is strongly recommended
Y	Participant timeline	13	Time schedule of enrolment, interventions (including any run-ins and washouts), assessments, and visits for participants. A schematic diagram is highly recommended (see Figure)

For peer review only - http://bmjopen.bmj.com/site/about/guidelines.xhtml

Y	Sample size	14	Estimated number of participants needed to achieve study objectives and how it was determined, including clinical and statistical assumptions supporting any sample size calculations
Y	Recruitment	15	Strategies for achieving adequate participant enrolment to reach target sample size
	**Methods: Assignment of interventions (for controlled trials)**		
	Allocation:		
Y	Sequence generation	16a	Method of generating the allocation sequence (eg, computer-generated random numbers), and list of any factors for stratification. To reduce predictability of a random sequence, details of any planned restriction (eg, blocking) should be provided in a separate document that is unavailable to those who enrol participants or assign interventions
NA	Allocation concealment mechanism	16b	Mechanism of implementing the allocation sequence (eg, central telephone; sequentially numbered, opaque, sealed envelopes), describing any steps to conceal the sequence until interventions are assigned
Y	Implementation	16c	Who will generate the allocation sequence, who will enrol participants, and who will assign participants to interventions
NA	Blinding (masking)	17a	Who will be blinded after assignment to interventions (eg, trial participants, care providers, outcome assessors, data analysts), and how
NA		17b	If blinded, circumstances under which unblinding is permissible, and procedure for revealing a participant's allocated intervention during the trial
	**Methods: Data collection, management, and analysis**		
Y	Data collection methods	18a	Plans for assessment and collection of outcome, baseline, and other trial data, including any related processes to promote data quality (eg, duplicate measurements, training of assessors) and a description of study instruments (eg, questionnaires, laboratory tests) along with their reliability and validity, if known. Reference to where data collection forms can be found, if not in the protocol

For peer review only - http://bmjopen.bmj.com/site/about/guidelines.xhtml

NA		18b	Plans to promote participant retention and complete follow-up, including list of any outcome data to be collected for participants who discontinue or deviate from intervention protocols
NA	Data management	19	Plans for data entry, coding, security, and storage, including any related processes to promote data quality (eg, double data entry; range checks for data values). Reference to where details of data management procedures can be found, if not in the protocol
Y	Statistical methods	20a	Statistical methods for analysing primary and secondary outcomes. Reference to where other details of the statistical analysis plan can be found, if not in the protocol
NA		20b	Methods for any additional analyses (eg, subgroup and adjusted analyses)
NA		20c	Definition of analysis population relating to protocol non-adherence (eg, as randomised analysis), and any statistical methods to handle missing data (eg, multiple imputation)
	**Methods: Monitoring**		
NA	Data monitoring	21a	Composition of data monitoring committee (DMC); summary of its role and reporting structure; statement of whether it is independent from the sponsor and competing interests; and reference to where further details about its charter can be found, if not in the protocol. Alternatively, an explanation of why a DMC is not needed
NA		21b	Description of any interim analyses and stopping guidelines, including who will have access to these interim results and make the final decision to terminate the trial
NA	Harms	22	Plans for collecting, assessing, reporting, and managing solicited and spontaneously reported adverse events and other unintended effects of trial interventions or trial conduct
NA	Auditing	23	Frequency and procedures for auditing trial conduct, if any, and whether the process will be independent from investigators and the sponsor
	**Ethics and dissemination**		

Y	Research ethics approval	24	Plans for seeking research ethics committee/institutional review board (REC/IRB) approval
NA	Protocol amendments	25	Plans for communicating important protocol modifications (eg, changes to eligibility criteria, outcomes, analyses) to relevant parties (eg, investigators, REC/IRBs, trial participants, trial registries, journals, regulators)
Y	Consent or assent	26a	Who will obtain informed consent or assent from potential trial participants or authorised surrogates, and how (see Item 32)
NA		26b	Additional consent provisions for collection and use of participant data and biological specimens in ancillary studies, if applicable
NA	Confidentiality	27	How personal information about potential and enrolled participants will be collected, shared, and maintained in order to protect confidentiality before, during, and after the trial
Y	Declaration of interests	28	Financial and other competing interests for principal investigators for the overall trial and each study site
NA	Access to data	29	Statement of who will have access to the final trial dataset, and disclosure of contractual agreements that limit such access for investigators
NA	Ancillary and post-trial care	30	Provisions, if any, for ancillary and post-trial care, and for compensation to those who suffer harm from trial participation
Y	Dissemination policy	31a	Plans for investigators and sponsor to communicate trial results to participants, healthcare professionals, the public, and other relevant groups (eg, via publication, reporting in results databases, or other data sharing arrangements), including any publication restrictions
Y		31b	Authorship eligibility guidelines and any intended use of professional writers
NA		31c	Plans, if any, for granting public access to the full protocol, participant-level dataset, and statistical code
	**Appendices**		

For peer review only

NA	Informed consent materials	32	Model consent form and other related documentation given to participants and authorised surrogates
NA	Biological specimens	33	Plans for collection, laboratory evaluation, and storage of biological specimens for genetic or molecular analysis in the current trial and for future use in ancillary studies, if applicable

*It is strongly recommended that this checklist be read in conjunction with the SPIRIT 2013 Explanation & Elaboration for important clarification on the items. Amendments to the protocol should be tracked and dated. The SPIRIT checklist is copyrighted by the SPIRIT Group under the Creative Commons "Attribution-NonCommercial-NoDerivs 3.0 Unported" license.

For peer review only

BMJ Open

Increasing the Reach of Evidence-Based Interventions for Weight Management and Diabetes Prevention Among Medicaid Patients: Study Protocol for a Pilot Sequential Multiple Assignment Randomized Trial.

Journal:	*BMJ Open*
Manuscript ID	bmjopen-2023-075157.R2
Article Type:	Protocol
Date Submitted by the Author:	29-Sep-2023
Complete List of Authors:	Schlechter, Chelsey; University of Utah Health, Population Health Sciences; University of Utah Health Huntsman Cancer Institute<br>Del Fiol, Guilherme; University of Utah, Department of Biomedical Informatics<br>Jones, Dusti; University of Utah Health, Department of Population Health Sciences; University of Utah Health Huntsman Cancer Institute<br>Orleans, Brian; University of Utah Health Huntsman Cancer Institute<br>Gibson, Bryan; University of Utah, Department of Biomedical Informatics<br>Nahum-Shani, Inbal; University of Michigan<br>Maxfield, Ellen; University of Utah Health Care, Osher Center for Integrative Health; University of Utah Health, Department of Family & Preventative Medicine<br>Locke, Amy; University of Utah Health, Osher Center for Integrative Health; University of Utah Health, Department of Family & Preventive Medicine<br>Cornia, Ryan; University of Utah, Department of Biomedical Informatics<br>Bradshaw, Richard; University of Utah, Department of Biomedical Informatics<br>Wirth, Jennifer; University of Utah Health Huntsman Cancer Institute<br>Jaggers, Shanna; University of Utah Health Huntsman Cancer Institute<br>Lam, Cho; University of Utah Health, Department of Population Health Sciences; University of Utah Health Huntsman Cancer Institute<br>Wetter, David; University of Utah Health, Department of Population Health Sciences; University of Utah Health Huntsman Cancer Institute
**Primary Subject Heading**:	Public health
Secondary Subject Heading:	Evidence based practice, Diabetes and endocrinology
Keywords:	PUBLIC HEALTH, Obesity, Health Equity, Health informatics < BIOTECHNOLOGY & BIOINFORMATICS, Telemedicine < BIOTECHNOLOGY & BIOINFORMATICS

SCHOLARONE™
Manuscripts

Authors: Chelsey R. Schlechter,[1,2+] Guilherme Del Fiol,[3] Dusti R. Jones,[1,2] Brian Orleans,[2] Bryan Gibson,[3] Inbal Nahum-Shani,[4] Ellen Maxfield,[5,6] Amy Locke,[5,6] Ryan Cornia,[3] Richard Bradshaw,[3] Jennifer Wirth,[2] Shanna J. Jaggers,[2] Cho Y. Lam,[1,2] David W. Wetter[1,2]

[1]Department of Population Health Sciences, University of Utah

[2]Huntsman Cancer Institute, University of Utah

[3]Department of Biomedical Informatics, University of Utah

[4]Institute for Social Research, University of Michigan

[5]Department of Family & Preventative Medicine, University of Utah

[6]Osher Center for Integrative Health, University of Utah

[+] Corresponding Author

Corresponding author's email address: Chelsey.schlechter@hci.utah.edu

Title: Increasing the Reach of Evidence-Based Interventions for Weight Management and Diabetes Prevention Among Medicaid Patients: Study Protocol for a Pilot Sequential Multiple Assignment Randomized Trial.

Abstract

Introduction: Over 40% of US adults meet criteria for obesity, a major risk factor for chronic disease. Obesity disproportionately impacts populations that have been historically marginalized (e.g., low socioeconomic status, rural, some racial/ethnic minority groups). Evidence-based interventions (EBIs) for weight management exist but reach less than 3% of eligible individuals. The aims of this pilot randomized controlled trial are to evaluate feasibility and acceptability of dissemination strategies designed to increase reach of EBIs for weight management.

Methods and analysis: This study is a two-phase, Sequential Multiple Assignment Randomized Trial, conducted with 200 Medicaid patients. In Phase 1, patients will be individually randomized to single text message (TM1) or multiple text messages (TM+). Phase 2 is based on treatment response. Patients who enroll in the EBI within 12 weeks of exposure to Phase 1 (i.e., responders) receive no further

For peer review only

interventions. Patients in TM1 who do not enroll in the EBI within 12 weeks of exposure (i.e., TM1 non-responders) will be randomized to either TM1-Continued (i.e., no further TM) or TM&MAPS (i.e., no further TM, up to 2 Motivation And Problem Solving [MAPS] navigation calls) over the next 12 weeks. Patients in TM+ who do not enroll in the EBI (i.e., TM+ non-responders) will be randomized to either TM+Continued (i.e., monthly text messages) or TM+&MAPS (i.e., monthly text messages, plus up to 2 MAPS calls) over the next 12 weeks. Descriptive statistics will be used to characterize feasibility (e.g., proportion of patients eligible, contacted, and enrolled in the trial) and acceptability (e.g., participant opt-out, participant engagement with dissemination strategies, EBI Reach [i.e., the proportion of participants who enroll in EBI], adherence, effectiveness).

Ethics and dissemination: Study protocol was approved by the University of Utah Institutional Review Board (#00139694). Results will be disseminated through study partners and peer-reviewed publications.

Strengths and limitations of this study

- Uses an innovative SMART design to optimize adaptive interventions.
- Potential reach and sustainability are enhanced by using dissemination strategy modalities (i.e., cellphone-based voice and text) that are ubiquitous among adults in the United States, including among historically marginalized populations, and are already utilized by payers and healthcare systems.
- Participant population was drawn from a single healthcare system in a single state.

Introduction

Approximately 40% of U.S. adults meet criteria for obesity (BMI ≥30),(1) and this proportion is expected to rise to nearly 50% in the next decade.(2) Obesity is a leading risk factor for multiple chronic diseases, including at least 13 different cancers,(3, 4, 5) Type 2 diabetes,(6, 7) cardiovascular disease,(8, 9) and premature death.(10) Additionally, obesity plays a critical role in health inequities, disproportionately impacting low socioeconomic status (SES), rural, and many racial/ethnic minority populations.(11, 12, 13, 14, 15) For example, individuals with lower levels of education have higher

For peer review only

obesity rates compared to those with higher levels of education.(16) Recent studies have found obesity rates of 41% among rural U.S. adults versus 32% among urban residents, and that rising BMI among rural populations has been a principal contributor to the obesity epidemic(17, 18) over the last 30 years. Finally, obesity is more prevalent among Latino adults compared to non-Latino White adults (45.6% and 41.4%, respectively).(15)

Evidence-based weight management and diabetes prevention programs. Recommendations from the United States Preventive Services Task Force and others highlight that adults with obesity should engage in comprehensive, multicomponent behavioral interventions addressing weight, diet, and physical activity.(19, 20, 21, 22) Components of these behavioral evidence-based interventions (EBIs) include improving diet and nutrition, increasing physical activity, and behavioral strategies such as goal setting and self-monitoring.(23) The Diabetes Prevention Program (DPP) is among the most well-known EBIs for weight management, with substantial evidence for weight loss, increased physical activity, decreased daily energy intake, and decreased incidence of Type 2 diabetes.(24) The DPP can be delivered via in-person or digital modalities, and there are currently over 1800 Centers for Disease Control and Prevention (CDC) recognized DPP programs across the U.S.(25) Despite their effectiveness, DPPs are grossly underutilized,(26) reaching only 3% of eligible individuals.(27) Furthermore, there are substantial disparities in DPP access (14.6% of rural counties contain a DPP site vs. 48.4% for urban counties)(28) and engagement among historically marginalized populations (e.g., low SES, Latino).(26, 29) Barriers to participation for in-person delivered DPPs among underserved populations include the need to travel long distances, and the lack of transportation, financial resources, childcare, and time. Fortunately, digital DPP delivery modalities such as the telephone, internet, and smartphones have yielded weight loss and other outcomes comparable to in-person DPPs, with greater engagement and lower attrition in digital programs.(24, 26, 30, 31, 32, 33, 34) Consistent with those findings, the Community Preventive Services Task Force concluded that "Technology-Supported Multicomponent Coaching or Counseling Interventions" are recommended for both weight loss and weight loss maintenance,(20) and that technology-supported programs may increase access among populations that have transportation issues,

For peer review only

unusual work schedules, or are rural. Similarly, the National Academy of Medicine report on obesity treatment identified key research recommendations that included improving access, employing technology to increase reach, and reducing health disparities.(21)

Dissemination Strategies to Increase EBI Reach. Dissemination strategies are designed to increase the reach of EBIs by increasing individual motivation and capability to access and engage with the interventions.(35, 36) Many strategies to increase the reach of weight management EBIs rely on dissemination strategies at the point of care (e.g., provider referral to EBI).(37, 38, 39) However, other potent strategies for increasing reach include proactive, population health management approaches that can remove the burden from clinical staff to offer the referral, and can also reach out to all appropriate patients without the need for a clinical visit to trigger the referral offer. Population health management approaches have increased referrals to EBIs for weight management(27, 37) and enrollment among eligible participants.(40, 41)

Digital and telehealth dissemination strategies such as text messaging and phone-based health coaching are promising approaches to improve dissemination of EBIs for weight management, and have near ubiquitous reach given 97% of Americans own a cell phone, including 97% of individuals with income less than $30,000 per year, 94% of rural individuals, and nearly 100% of Hispanic individuals.(42) Text messaging has been effective for improving numerous health behaviors (e.g., physical activity, nutrition, and tobacco cessation), and for improving attendance and compliance with behavioral interventions.(43, 44, 45, 46, 47, 48) Similarly, personalized support to address barriers to care via patient navigation has been shown to improve completion rates for cancer screening, increase adherence to medication, improve compliance to treatment and follow-up visits, improve biomarkers for diabetes, and increase enrollment in EBIs.(49, 50, 51, 52) However, there is limited research on the effectiveness of these approaches for increasing reach of EBIs for weight management among historically marginalized populations.

For peer review only

Adaptive interventions. Adaptive interventions contain a sequence of individually tailored decision rules that specify the intensity or type of intervention at critical points in the delivery of care.(53, 54, 55). Modifying intensity or type of intervention over time can improve outcomes if an individual is not responding, or may reduce costs and burden if intensive treatment is not necessary.(56) For example, a stepped care model (a type of adaptive intervention) can conserve scarce resources and reduce burden on patients. Sequential Multiple Assignment Randomized Trials (SMART(57, 58)) are a type of experimental design used to inform the dosage, type, delivery, and timing of adaptive interventions.

Aims and objectives. The overall aims of this pilot study are to evaluate the feasibility and acceptability of the dissemination strategies and study procedures. Feasibility will be measured by the proportion of people who were eligible, contacted, and randomized in the trial. Acceptability will be assessed by rate of participant opt-out, participant engagement with dissemination strategies, EBI Reach by randomization condition, and EBI adherence and effectiveness among participants who enroll in the EBI.

Methods and Analysis

The protocol for this pilot study has been reviewed and approved by the University of Utah Institutional Review Board (IRB). As study interventions were deemed no more than minimal risk, documentation of informed consent was waived for the project. All participants will be notified of the study and given the opportunity to opt-out of the project using a cover letter.

Data collection was planned to begin March 2022 and end in May 2023. The trial is registered at clinicaltrials.gov (NCT05666323). This manuscript adheres to the Standards Protocol Items: Recommendations for Interventional Trials (SPIRIT).

Settings and Participants.

Participants will be 200 adults 1) age ≥ 18, 2) BMI ≥ 30; 2) have had a primary care appointment within University of Utah Health (UHealth) within the past 12 months; 3) speak English or Spanish as their primary language; 4) have a current Utah address; and 5) are insured through Medicaid by the

For peer review only

University of Utah Health Plans. Exclusion criteria include 1) a diagnosis of Type 1 or Type 2 diabetes, 2) documented current pregnancy, and 3) no phone number listed in the EHR.

Study Design.

This trial is a two-phase, Sequential Multiple Assignment Randomized Trial (SMART) with 200 Medicaid patients from a single academic medical system (Figure 1). In the first phase, participants will be individually randomized to receive a single text message (TM1), or multiple text messages (TM+) offering free enrollment in a digital EBI for weight management.

Phase 2 dissemination strategies are randomized based on prior dissemination strategy response. Participants in TM1 who do not opt out or enroll in EBI by 12 weeks will be randomized to either TM1-Continued (no further TM) or TM1 & MAPS (no further TM and up to 2 Motivation And Problem Solving [MAPS] calls from a patient navigator). Participants in TM+ who do not opt out or enroll in the EBI by 12 weeks will be randomized to either TM+ Continued (monthly text messages for the next 12 weeks) or TM+ & MAPS (monthly text messages for the next 12 weeks, plus up to 2 MAPS calls from a patient navigator). Descriptions of participant interventions are listed in Table 1.

Table 1: Randomization Condition Description

	Randomization Condition	Description
**Phase 1**		
	Single text message (TM1)	Participants receive a single text message that includes a motivational message, the EBI phone number, and a two-touch response that directly connects interested participants to EBI enrollment.
	Multiple text message (TM+)	Participants receive messages once per week for the first 2 weeks, then 1 message every 3 weeks for the remaining 10 weeks (up to 5 texts total). The text messages include a motivational message, the EBI phone number, and a simple two-touch response that directly connects interested participants to EBI enrollment.
**Phase 2**		
For TM1 non-responding participants only.	Single text message continued (TM1-Cont)	Participants receive no additional text messages.
	Single text message & Motivation And Problem Solving (MAPS) (TM1 & MAPS)	Participants receive no additional text messages and receive up to 2 MAPS counselling calls from a health coach.

For peer review only

For TM+ non-responding participants only.	Multiple text message continued (TM+-Cont)	Participants receive text messages every 4 weeks for the next 12 weeks (up to 3 text messages total).
	Multiple text message & MAPS (TM+ & MAPS)	Participants receive text messages every 4 weeks for the next 12 weeks (up to 3 text messages total) plus up to 2 MAPS counselling calls from a health coach.

Dissemination Strategies

Text Messaging. Participants will receive bidirectional Health Insurance Portability and Accountability Act (HIPAA) compliant text messages that include a brief motivational message, the opportunity to enroll in the EBI, and the opportunity to opt-out of receiving further dissemination strategies.

Motivation And Problem Solving (MAPS) Health Coaching. Participants will receive up to two brief calls from health coaches trained in using MAPS, a coaching/counselling approach designed to help participants address the larger context in which behavior change occurs, identify and address barriers to change, and motivate participants towards a long-term goal of change. MAPS is a holistic, dynamic behavioral approach that integrates two empirically validated approaches (motivational interviewing(59, 60) and problem solving/social cognitive strategies(61, 62, 63)) into a coordinated intervention with demonstrated efficacy for addressing the entire process of considering behavior change, attempting to change, and maintaining change.(50, 63, 64, 65, 66) MAPS employs a “wellness” approach that in addition to focusing on the target behavior, provides practical advice and connections to additional services whenever possible for life events, stressors, and concerns that impact the lives of rural and other historically marginalized individuals (e.g., isolation, stress, depression, financial barriers, etc.,). MAPS is based on substantial evidence that motivation fluctuates frequently and rapidly, and as such, is designed to fluidly respond to those changes.(67, 68, 69, 70) MAPS health coaching calls will be delivered by two bilingual health coaches on the research team with at least 20 hours of training in MAPS delivery.

Digital EBI for Diabetes Prevention and Weight Management.

For peer review only

Participants who enroll in the EBI will receive either a digital DPP or digital weight management program based on criteria for meeting prediabetes. Participants will be evaluated for risk of prediabetes using the American Diabetes Association (ADA) and CDC Prediabetes Risk Test(71) at the time of enrolling in the EBI. The test uses only self-report health trait data (e.g., family history, height, weight, physical activity level, health history of hypertension and gestational diabetes) and does not require testing data (i.e., hemoglobin A1C or cholesterol levels). Participants who meet criteria for prediabetes based on the risk test will be triaged into a digital DPP program; participants who do not meet criteria will be triaged to a digital weight management program. Both programs will be delivered by EBI vendor, IncentaHEALTH. Based on national data on eligibility for the DPP among individuals with obesity and data from the EBI provider, we estimate that ~50% of participants will be triaged to DPP and ~50% to weight management.(72)

The digital DPP offered to study participants is a CDC fully recognized program. The digital weight management program, for all participants who do not meet criteria for the DPP, uses the same general components as the DPP. Both programs are designed to increase intake of fruits, vegetables, lean protein and complex carbohydrates while monitoring portion size. Physical activity recommendations include cardiovascular and strength training, with a goal of approximately 30 minutes 5-6 days/week. Both programs are offered in English and Spanish, based on Social Cognitive Theory, and provide personalized health coaching. Both include a website, a Bluetooth wireless scale for weight monitoring, daily email/text message support, easy to follow video exercise plans, customized meal plans, grocery lists, incorporate skill building, and incorporate social accountability. Participants have unlimited access to Health Coaches, who have at least a Bachelor's degree, along with certifications, in personal training, nutrition, or wellness.

Recruitment.

The flow of participants through the study is described in Figure 2. Participants who meet inclusion criteria will be identified from the University of Utah Health (UHealth) Electronic Data Warehouse (EDW). Participant demographic, preferred language, and relevant health data, as well as

For peer review only

participant contact information (i.e., phone number, address, email address) will be extracted from the EDW for all eligible participants. Participants who meet exclusionary criteria will be removed from the eligible population. We will use quota sampling from the eligible population to achieve a stratified random sample aimed to offer balance in the marginal distributions of key participant demographic characteristics. These margins include 1) male/female sex (1:1 ratio), 2) urban/rural according to participant zip code and the 2010 Rural Urban Commuting Area (RUCA) codes (>= 4 defined as rural) (1:1 ratio), and 3) Latino with English as preferred language, Latino with Spanish as preferred language, and non-Latino with English as preferred language (1:1:3 ratio).

Sampled patients randomized to TM1 or TM+ will be sent an e-mail informing them that they will be receiving communications from UHealth about the availability of a digital EBI for weight management. The e-mail will provide patients with information about how they can opt-out if they do not wish to receive these communications. Patients who do not have an email in the EHR will be sent the opt-out information via mail. Patients who opt-out of the study receive no further interventions.

Randomization and Blinding.

In Phase 1 (Figure 1), participants will be randomly assigned to TM1 or TM+ in a 1:1 ratio. In Phase 2 (Figure 1), participants who opt out or who enroll in the EBI will receive no further dissemination strategies and will not be re-randomized. Participants in TM1 did not opt-out and who did not enroll in the EBI will be randomized to TM1-Cont or TM1 & MAPS at a ratio of 1:1. Participants in TM+ who did not opt-out and did not enroll in the EBI will be randomized to TM+-Cont or TM+ & MAPS at a ratio of 1:1. All randomization sequences will be generated via SAS 9.4 to consist of random permuted blocks with random block sizes, thus ensuring balance in the allocation over time.

Data Collection and Outcomes.

Participant demographic and baseline health data (e.g., age, gender, BMI, pregnancy status, medical codes for Type 1 and Type 2 diabetes, zip code, preferred language, race/ethnicity) will be obtained from the UHealth EDW.

For peer review only

Feasibility outcomes. Feasibility will be assessed using the proportion of participants who were eligible, contacted, and enrolled in the trial.

Acceptability outcomes. Acceptability will be assessed using participant opt-out, participant engagement with dissemination strategies, EBI reach, and EBI effectiveness. Participant opt-out is assessed by the absolute number and proportion of eligible participants who opt-out during Phase 1 and Phase 2. Participant engagement will be assessed as 1) the number and proportion of participants who respond to text messages with a response other than opt-out, 2) the number and proportion of eligible participants who talk to a MAPS health coach, 3) the number and proportion of eligible participants accept connection to the EBI, and 4) the number and proportion of participants who talk to the EBI personnel. EBI reach is assessed by the number and proportion of eligible participants who officially enroll in the EBI. Enrollment is defined in accordance with the CDC definition for recognized virtual programs, which consists of the participant setting a password for the program. Enrollment will be obtained from the EBI vendor (IncentaHEALTH). EBI adherence and effectiveness are measured by participant adherence to the EBI components, percentage of body-weight lost, and change in BMI, and will be collected for 12 weeks following EBI enrollment. Adherence and effectiveness data will be obtained from the EBI vendor (IncentaHEALTH)

Patient and Public Involvement.

This study was developed and implemented in collaboration with the University of Utah Wellness and Integrative Health Program, which supports population health initiatives through integration of wellness services into patient care and health plan offerings. The program currently manages lifestyle behavior change programs (including the Diabetes Prevention Program) for UHealth patients and University of Utah faculty, staff, and students. The dissemination strategies, the feasibility and acceptability outcomes, and study procedures were developed in collaboration with the University of Utah Wellness and Integrative Health Program and investigators at the University of Utah. Patients were not directly involved in the development of this pilot study.

Ethics and dissemination.

For peer review only

All procedures performed in studies involving human participants will be conducted in accordance with the ethical standards of the institutional and/or national research committee and with the 1964 Helsinki declaration and its later amendments or comparable ethical standards. The protocol for this study was approved by the University of Utah Institutional Review Board (00139694). Materials used to conduct the study are not currently publicly available. Materials may be requested by emailing the corresponding author. Study results will be dissemination via peer reviewed publications and manuscripts, as well as to the health system and community partners via lay reports and presentations.

Data Analysis Plan.

Our primary study aim is to evaluate the acceptability and feasibility of dissemination strategies to increase of EBIs for weight management.

Primary Analyses Phase 1. The analysis of acceptability and feasibility at 12 weeks will include all participants enrolled in the study. We will use descriptive statistics (mean, standard deviation (SD), confidence interval) to evaluate each indicator of feasibility and acceptability by dissemination strategies (i.e., TM1, TM+).

Primary Analyses Phase 2. The analysis of feasibility and acceptability at 24-weeks will be conducted using only data from those participants who did not opt out or enroll in the EBIs by 12 weeks (i.e., non-responders). We will use descriptive statistics to evaluate each indicator of feasibility and acceptability by dissemination strategies (i.e., TM1-Cont, TM1 & MAPS, TM+-Cont, TM+ & MAPS).

Power Analysis.

The sample size (N = 200) for the current project was determined based on feasibility of sample size recruitment from a single Medicaid provider and completing project procedures in the study timeframe. As such, power calculations were not conducted for this pilot trial.

Strengths and Limitations.

The proposed pilot study uses an innovative SMART design to optimize adaptive interventions to increase reach of already existing EBIs for weight management among historically marginalized populations. Potential reach and sustainability are enhanced by using dissemination strategy modalities

For peer review only

(i.e., cellphone-based voice and text) that are ubiquitous among adults in the United States, including among historically marginalized populations, and are already utilized by payers and healthcare systems. The participant population was drawn from a single healthcare system in a single state which may limit generalizability of results. However, the proposed study population will represent a diverse sample based on sex, ethnicity, primary language, and rurality.

Conclusion

The proposed pilot project is a two-phase SMART with the objective of evaluating acceptability and feasibility of dissemination strategies designed to increase the reach of digital EBIs for weight management among Medicaid patients. Accomplishing the aims of the proposed study will provide critical data regarding the feasibility and acceptability of pragmatic and scalable dissemination strategies designed to increase the uptake of digital EBIs for obesity among historically marginalized populations, and inform the conduct of a future, fully powered randomized trial.

Registration details: The trial is registered at clinicaltrials.gov (NCT05666323).

Funding statement. This work was supported through the Ben B. and Iris M. Margolis Foundation, the National Center for Advancing Translational Sciences of the National Institutes of Health (UL1TR002538), the National Cancer Institute (P30CA042014), and the Huntsman Cancer Foundation.

Competing interests. The authors declare no competing interests.

Author contribution. DWW acquired financial support for the project. CRS, GDF, BO, BG, EM, AL, JW, CYL, RB, RC, SJ, IN and DWW contributed to study conceptualization. All authors contributed to the development or design of the methodology, dissemination strategies, study procedures, study outcomes, or analytic plan. CRS and DRJ drafted the original manuscript. All authors reviewed, edited, and approved the final manuscript.

Figure caption.

Figure 1: Sequential multiple assignment randomized trial (SMART) study design

For peer review only

Figure 2: Patient flow diagram

References

1. Hales CM, Carroll MD, Fryar CD, Ogden CL. Prevalence of Obesity and Severe Obesity Among Adults: United States, 2017–2018. NCHS Data Brief. 2020;no. 360(1-7).
2. Ward ZJ, Bleich SN, Cradock AL, Barrett JL, Giles CM, Flax C, et al. Projected U.S. State-Level Prevalence of Adult Obesity and Severe Obesity. N Engl J Med. 2019;381(25):2440-50.
3. Islami F, Goding Sauer A, Miller KD, Siegel RL, Fedewa SA, Jacobs EJ, et al. Proportion and number of cancer cases and deaths attributable to potentially modifiable risk factors in the United States. CA: A Cancer Journal for Clinicians. 2018;68(1):31-54.
4. Islami F, Goding Sauer A, Gapstur SM, Jemal A. Proportion of Cancer Cases Attributable to Excess Body Weight by US State, 2011-2015. JAMA Oncology. 2019;5(3):384-92.
5. McTiernan A, Friedenreich CM, Katzmarzyk PT, Powell KE, Macko R, Buchner D, et al. Physical Activity in Cancer Prevention and Survival: A Systematic Review. Med Sci Sports Exerc. 2019;51(6):1252-61.
6. Verma S, Hussain ME. Obesity and diabetes: An update. Diabetes Metab Syndr. 2017;11(1):73-9.
7. Mokdad AH, Ford ES, Bowman BA, Dietz WH, Vinicor F, Bales VS, et al. Prevalence of obesity, diabetes, and obesity-related health risk factors, 2001. Jama. 2003;289(1):76-9.
8. Powell-Wiley TM, Poirier P, Burke LE, Després J-P, Gordon-Larsen P, Lavie CJ, et al. Obesity and cardiovascular disease: a scientific statement from the American Heart Association. Circulation. 2021;143(21):e984-e1010.
9. Khan SS, Ning H, Wilkins JT, Allen N, Carnethon M, Berry JD, et al. Association of Body Mass Index With Lifetime Risk of Cardiovascular Disease and Compression of Morbidity. JAMA Cardiol. 2018;3(4):280-7.
10. Hruby A, Manson JE, Qi L, Malik VS, Rimm EB, Sun Q, et al. Determinants and Consequences of Obesity. Am J Public Health. 2016;106(9):1656-62.
11. AHRQ. 2018 National Healthcare Quality and Disparities. Rockville, MD: Agency for Healthcare Research and Quality; September 2019. Contract No.: AHRQ Publication No. 19-0070-EF.
12. Clarke AR, Goddu AP, Nocon RS, Stock NW, Chyr LC, Akuoko JAS, et al. Thirty years of disparities intervention research: what are we doing to close racial and ethnic gaps in health care? Med Care. 2013;51(11):1020-6.
13. Crosby RA, Wendel ML, Vanderpool RC, Casey BR, editors. Rural populations and health: determinants, disparities, and solutions. San Francisco, CA: Jossey-Bass; 2012.
14. National Center for Health Statistics. Health, United States, 2011: With Special Feature on Socioeconomic Status and Health. Hyattsville, MD: National Center for Health Statistics (US); 2012.
15. Akinbami LJ, Chen TC, Davy O, Ogden CL, Fink S, Clark J, et al. National Health and Nutrition Examination Survey, 2017-March 2020 Prepandemic File: Sample Design, Estimation, and Analytic Guidelines. Vital Health Stat 1. 2022(190):1-36.

For peer review only

16. Stierman B, Afful J, Carroll MD, Chen T-C, Davy O, Fink S, et al. National Health and Nutrition Examination Survey 2017–March 2020 prepandemic data files development of files and prevalence estimates for selected health outcomes. 2021.
17. NCD Risk Factor Collaboration. Rising rural body-mass index is the main driver of the global obesity epidemic in adults. Nature. 2019;569(7755):260.
18. Wen M, Fan JX, Kowaleski-Jones L, Wan N. Rural–urban disparities in obesity prevalence among working age adults in the United States: exploring the mechanisms. American Journal of Health Promotion. 2018;32(2):400-8.
19. Jensen MD, Ryan DH, Apovian CM, Ard J, Comuzzie AG, Donato KA, et al. 2013 AHA/ACC/TOS guideline for the management of overweight and obesity in adults: a report of the American College of Cardiology/American Heart Association task force on practice guidelines and the obesity society. Circulation. 2014;129(25 Suppl 2):S102-S38.
20. Community Preventive Services Task Force. Obesity: Technology-Supported Multicomponent Coaching or Counseling Interventions to Maintain Weight Loss. Retrieved from https://www.thecommunityguide.org/findings/obesity-technology-supported-multicomponent-coaching-or-counseling-interventions-maintain.
21. Heymsfield S, Aronne LJ, Eneli I, Kumar RB, Michalsky M, Walker E, et al. Clinical Perspectives on Obesity Treatment: Challenges, Gaps, and Promising Opportunities. NAM Perspectives. 2018.
22. LeBlanc ES, Patnode CD, Webber EM, Redmond N, Rushkin M, O'Connor EA. Behavioral and pharmacotherapy weight loss interventions to prevent obesity-related morbidity and mortality in adults: Updated evidence report and systematic review for the US Preventive Services Task Force. Jama. 2018;320(11):1172-91.
23. Curry SJ, Krist AH, Owens DK, Barry MJ, Caughey AB, Davidson KW, et al. Behavioral weight loss interventions to prevent obesity-related morbidity and mortality in adults: US preventive services task force recommendation statement. Jama. 2018;320(11):1163-71.
24. Dunkley AJ, Bodicoat DH, Greaves CJ, Russell C, Yates T, Davies MJ, et al. Diabetes prevention in the real world: effectiveness of pragmatic lifestyle interventions for the prevention of type 2 diabetes and of the impact of adherence to guideline recommendations: a systematic review and meta-analysis. Diabetes care. 2014;37(4):922-33.
25. CDC. Diabetes Prevention Recognition Program – Registry of Recognized Organizations [Available from: https://nccd.cdc.gov/DDT_DPRP/Registry.aspx.
26. Ali MK, Bullard KM, Imperatore G, Benoit SR, Rolka DB, Albright AL, et al. Reach and use of diabetes prevention services in the United States, 2016-2017. JAMA network open. 2019;2(5):e193160-e.
27. Alva ML, Chakkalakal RJ, Moin T, Galaviz KI. The Diabetes Prevention Gap And Opportunities To Increase Participation In Effective Interventions. Health Aff (Millwood). 2022;41(7):971-9.
28. Ariel-Donges AH, Gordon EL, Dixon BN, Eastman AJ, Bauman V, Ross KM, et al. Rural/urban disparities in access to the National Diabetes Prevention Program. Translational behavioral medicine. 2019.
29. Ritchie ND, Christoe-Frazier L, McFann KK, Havranek EP, Pereira RI. Effect of the National Diabetes Prevention Program on Weight Loss for English- and Spanish-Speaking Latinos. American Journal of Health Promotion. 2017;32(3):812-5.

For peer review only

30. Moin T, Damschroder LJ, AuYoung M, Maciejewski ML, Havens K, Ertl K, et al. Results From a Trial of an Online Diabetes Prevention Program Intervention. American Journal of Preventive Medicine. 2018;55(5):583-91.
31. Mudaliar U, Zabetian A, Goodman M, Echouffo-Tcheugui JB, Albright AL, Gregg EW, et al. Cardiometabolic risk factor changes observed in diabetes prevention programs in US settings: a systematic review and meta-analysis. PLoS medicine. 2016;13(7).
32. Taetzsch A, Gilhooly CH, Bukhari A, Das SK, Martin E, Hatch AM, et al. Development of a Videoconference-Adapted Version of the Community Diabetes Prevention Program, and Comparison of Weight Loss With In-Person Program Delivery. Military Medicine. 2019;184(11-12):647-52.
33. Tice J, Chapman R, Shore KK, Seidner M, Ollendorf D, Weissberg J, et al. Diabetes Prevention Programs: effectiveness and value: final evidence report and meeting summary. Institute for Clinical and Economic Review; 2018.
34. Kim SE, Sweet CMC, Gibson E, Madero EN, Rubino B, Morrison J, et al. Evaluation of a digital diabetes prevention program adapted for the Medicaid population: study design and methods for a non-randomized, controlled trial. Contemporary clinical trials communications. 2018;10:161-8.
35. Lomas J. Diffusion, dissemination, and implementation: who should do what? Ann N Y Acad Sci. 1993;703:226-35; discussion 35-7.
36. McCormack L, Sheridan S, Lewis M, Boudewyns V, Melvin CL, Kistler C, et al. Communication and dissemination strategies to facilitate the use of health-related evidence. Evid Rep Technol Assess (Full Rep). 2013(213):1-520.
37. Holliday CS, Williams J, Salcedo V, Kandula NR. Clinical Identification and Referral of Adults With Prediabetes to a Diabetes Prevention Program. Prev Chronic Dis. 2019;16:E82.
38. Nhim K, Khan T, Gruss S, Wozniak G, Kirley K, Schumacher P, et al. Facilitators to referrals to CDC's National Diabetes Prevention Program in primary care practices and pharmacies: DocStyles 2016-2017. Prev Med. 2021;149:106614.
39. Kirley K, Khan T, Aquino G, Brown A, Meier S, Chambers N, et al. Using a certified electronic health record technology platform to screen, test and refer patients with prediabetes. JAMIA Open. 2021;4(4):ooab101.
40. Porter G, Michaud TL, Schwab RJ, Hill JL, Estabrooks PA. Reach Outcomes and Costs of Different Physician Referral Strategies for a Weight Management Program Among Rural Primary Care Patients: Type 3 Hybrid Effectiveness-Implementation Trial. JMIR Formative Research. 2021;5(10):e28622.
41. Wilson KE, Michaud TL, Almeida FA, Schwab RJ, Porter GC, Aquilina KH, et al. Using a population health management approach to enroll participants in a diabetes prevention trial: reach outcomes from the PREDICTS randomized clinical trial. Translational behavioral medicine. 2021;11(5):1066-77.
42. Center PR. Mobile Fact Sheet 2021 [Available from: https://www.pewresearch.org/internet/fact-sheet/mobile/.
43. Cole-Lewis H, Kershaw T. Text messaging as a tool for behavior change in disease prevention and management. Epidemiologic reviews. 2010:mxq004.

For peer review only

44. Gurol-Urganci I, de Jongh T, Vodopivec-Jamsek V, Atun R, Car J. Mobile phone messaging reminders for attendance at healthcare appointments. Cochrane Database of Systematic Reviews. 2013(12).
45. Head KJ, Noar SM, Iannarino NT, Harrington NG. Efficacy of text messaging-based interventions for health promotion: a meta-analysis. Social Science & Medicine. 2013;97:41-8.
46. Schwebel FJ, Larimer ME. Using text message reminders in health care services: A narrative literature review. Internet Interventions. 2018;13:82-104.
47. Vodopivec-Jamsek V, de Jongh T, Gurol-Urganci I, Atun R, Car J. Mobile phone messaging for preventive health care. The Cochrane Library. 2012.
48. USDHHS. Using Health Text Messages To Improve Consumer Health Knowledge, Behaviors, And Outcomes: An Environmental Scan. Rockville, MD: US Department of Health and Human Services; 2014.
49. McBrien KA, Ivers N, Barnieh L, Bailey JJ, Lorenzetti DL, Nicholas D, et al. Patient navigators for people with chronic disease: A systematic review. PloS one. 2018;13(2):e0191980-e.
50. Vinci C, Lam C, Schlechter CR, Shono Y, Vidrine JI, Wetter DW. Increasing treatment enrollment among smokers who are not motivated to quit: a randomized clinical trial. Translational Behavioral Medicine. 2021.
51. Peart A, Lewis V, Brown T, Russell G. Patient navigators facilitating access to primary care: a scoping review. BMJ open. 2018;8(3):e019252.
52. Valaitis RK, Carter N, Lam A, Nicholl J, Feather J, Cleghorn L. Implementation and maintenance of patient navigation programs linking primary care with community-based health and social services: a scoping literature review. BMC health services research. 2017;17(1):116.
53. Almirall D, Collins LM, Murphy SA. Introduction to adaptive interventions and SMART study design principles. COPTR Workshop on Adaptive Interventions 2011.
54. Bierman KL, Nix RL, Maples JJ, Murphy SA. Examining clinical judgment in an adaptive intervention design: The fast track program. Journal of Consulting and Clinical Psychology. 2006;74(3):468.
55. McKay JR. Is there a case for extended interventions for alcohol and drug use disorders? Addiction. 2005;100(11):1594-610.
56. Lei H, Nahum-Shani I, Lynch K, Oslin D, Murphy S. A" SMART" design for building individualized treatment sequences. Annual review of clinical psychology. 2012;8:21-48.
57. Lavori PW, Dawson R. Introduction to dynamic treatment strategies and sequential multiple assignment randomization. Clinical Trials. 2014:1740774514527651.
58. Murphy SA. An experimental design for the development of adaptive treatment strategies. Statistics in medicine. 2005;24(10):1455-81.
59. Miller WR, Rollnick S. Motivational Interviewing: Preparing People to Change Addictive Behavior. New York: The Guildford Press; 1991.
60. Miller WR, Rollnick S. Motivational Interviewing (2nd edition). New York: The Guilford Press; 2002.
61. Marlatt G, Donovan D, editors. Relapse prevention: Maintenance strategies in the treatment of addictive behaviors (2nd edition). New York: Guilford Press; 2005.
62. Witkiewitz K, Marlatt GA. Relapse prevention for alcohol and drug problems: That was Zen, this is Tao. American Psychologist. 2004;59(4):224-35.

For peer review only

63. Reitzel LR, Vidrine JI, Businelle MS, Kendzor DE, Costello TJ, Li Y, et al. Preventing postpartum smoking relapse among diverse low-income women: a randomized clinical trial. Nicotine Tob Res. 2010;12(4):326-35.
64. Correa-Fernández V, Díaz-Toro EC, Reitzel LR, Guo L, Chen M, Li Y, et al. Combined treatment for at-risk drinking and smoking cessation among Puerto Ricans: A randomized clinical trial. Addictive Behaviors. 2017;65:185-92.
65. McClure JB, Westbrook E, Curry SJ, Wetter DW. Proactive, motivationally enhanced smoking cessation counseling among women with elevated cervical cancer risk. Nicotine & Tobacco Research. 2005;7(6):881-9.
66. Wetter DW, Mazas C, Daza P, Nguyen L, Fouladi RT, Li Y, et al. Reaching and treating Spanish-speaking smokers through the National Cancer Institute's Cancer Information Service. A randomized controlled trial. Cancer. 2007;109(2 Suppl):406-13.
67. Conroy DE, Elavsky S, Hyde AL, Doerksen SE. The dynamic nature of physical activity intentions: a within-person perspective on intention-behavior coupling. Journal of Sport and Exercise Psychology. 2011;33(6):807-27.
68. Maher JP, Dunton GF. Within-day time-varying associations between motivation and movement-related behaviors in older adults. Psychology of Sport and Exercise. 2020;47:101522.
69. Vartanian LR, Pinkus RT, Smyth JM. Experiences of weight stigma in everyday life: Implications for health motivation. Stigma and Health. 2018;3(2):85-92.
70. Vidrine JI, Reitzel LR, Figueroa P, Velasquez MM, Mazas CA, Cinciripini PM, et al. Motivation And Problem Solving (MAPS): Motivationally-based skills training for treating substance use. Cogn Behav Pract. 2013;20(4):501-16.
71. Centers for Disease Control Prevention ADA. Predaibetes Risk Test [Available from: https://www.cdc.gov/diabetes/prevention/pdf/Prediabetes-Risk-Test-Final.pdf.
72. Bullard KM, Saydah SH, Imperatore G, Cowie CC, Gregg EW, Geiss LS, et al. Secular changes in US prediabetes prevalence defined by hemoglobin A1c and fasting plasma glucose: National Health and Nutrition Examination Surveys, 1999–2010. Diabetes Care. 2013;36(8):2286-93.

For peer review only

Figure 1: Study Design

Two Phase Sequential Multiple Assignment Randomized Trial (SMART)

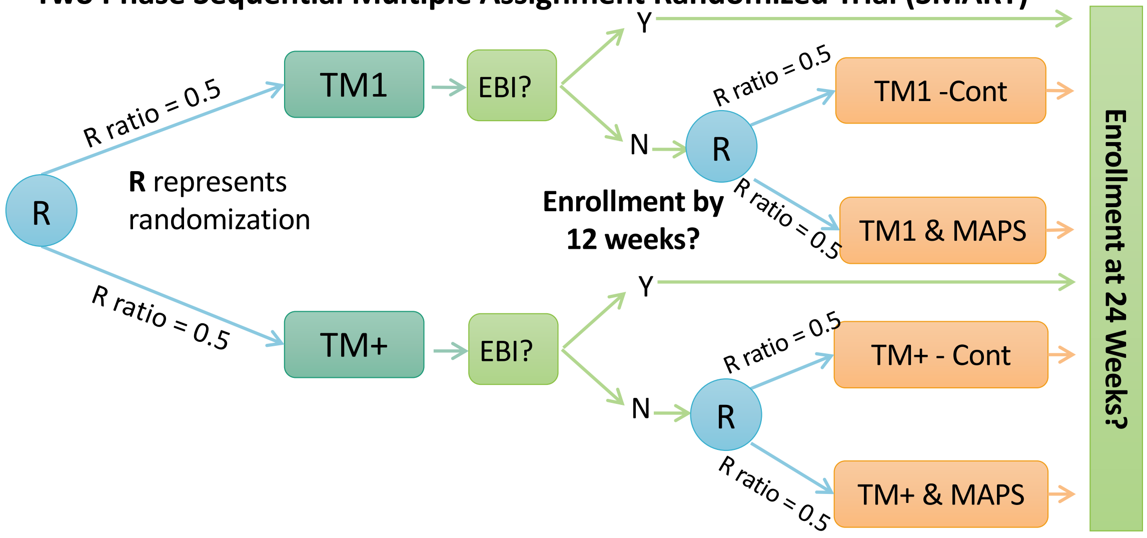

Phase 1 (1:1 ratio)

- **TM1** = Text Message 1
- **TM+** = Text Message +

Phase 2 (1:1 ratio)

- **TM1 Cont** = Continued TM1
- **TM1 & MAPS** = TM & Motivation And Problem Solving (MAPS)
- **TM+ Cont** = Continued TM
- **TM+ & MAPS** = TM and Motivation And Problem Solving (MAPS)

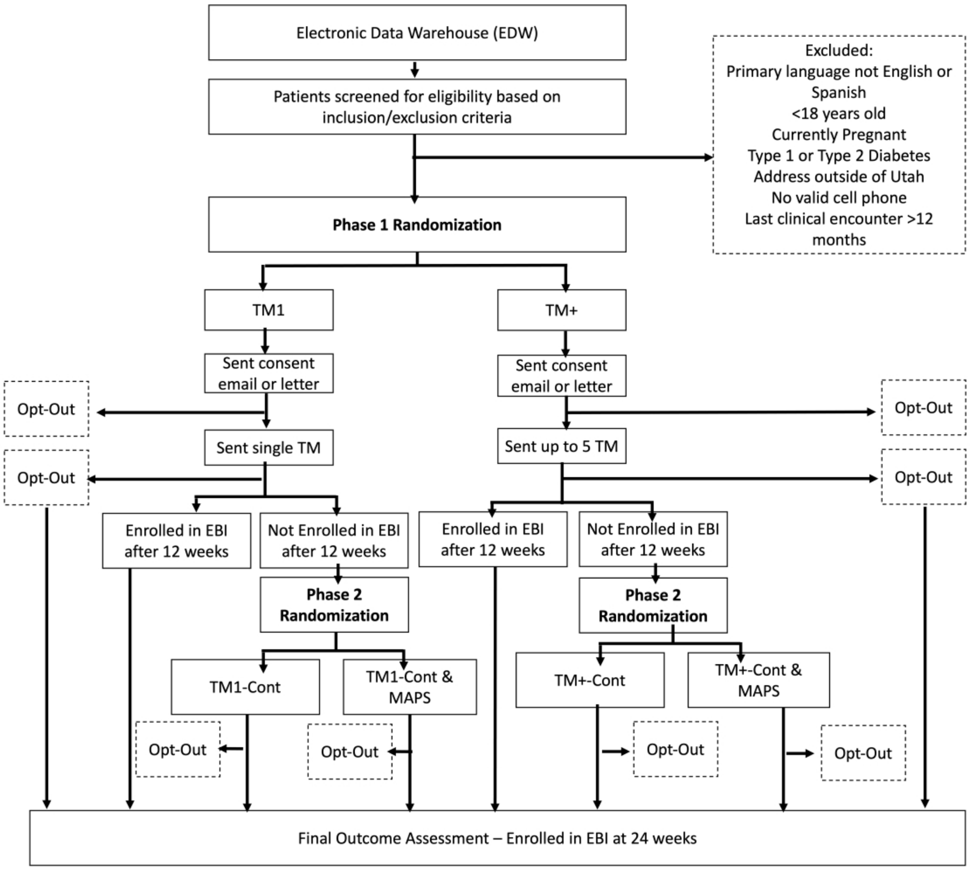

Figure 2: Consort Diagram

361x321mm (330 x 330 DPI)

SPIRIT

Standard Protocol Items: Recommendations for Interventional Trials

SPIRIT 2013 Checklist: Recommended items to address in a clinical trial protocol and related documents*

	Section/item	ItemNo	Description
	**Administrative information**		
Y	Title	1	Descriptive title identifying the study design, population, interventions, and, if applicable, trial acronym
Y	Trial registration	2a	Trial identifier and registry name. If not yet registered, name of intended registry
NA		2b	All items from the World Health Organization Trial Registration Data Set
NA	Protocol version	3	Date and version identifier
Y	Funding	4	Sources and types of financial, material, and other support
Y	Roles and responsibilities	5a	Names, affiliations, and roles of protocol contributors
Y		5b	Name and contact information for the trial sponsor
Y		5c	Role of study sponsor and funders, if any, in study design; collection, management, analysis, and interpretation of data; writing of the report; and the decision to submit the report for publication, including whether they will have ultimate authority over any of these activities
NA		5d	Composition, roles, and responsibilities of the coordinating centre, steering committee, endpoint adjudication committee, data management team, and other individuals or groups overseeing the trial, if applicable (see Item 21a for data monitoring committee)
	**Introduction**		
Y	Background and rationale	6a	Description of research question and justification for undertaking the trial, including summary of relevant studies (published and unpublished) examining benefits and harms for each intervention

For peer review only

Y		6b	Explanation for choice of comparators
Y	Objectives	7	Specific objectives or hypotheses
Y	Trial design	8	Description of trial design including type of trial (eg, parallel group, crossover, factorial, single group), allocation ratio, and framework (eg, superiority, equivalence, noninferiority, exploratory)
	**Methods: Participants, interventions, and outcomes**		
Y	Study setting	9	Description of study settings (eg, community clinic, academic hospital) and list of countries where data will be collected. Reference to where list of study sites can be obtained
Y	Eligibility criteria	10	Inclusion and exclusion criteria for participants. If applicable, eligibility criteria for study centres and individuals who will perform the interventions (eg, surgeons, psychotherapists)
Y	Interventions	11a	Interventions for each group with sufficient detail to allow replication, including how and when they will be administered
NA		11b	Criteria for discontinuing or modifying allocated interventions for a given trial participant (eg, drug dose change in response to harms, participant request, or improving/worsening disease)
NA		11c	Strategies to improve adherence to intervention protocols, and any procedures for monitoring adherence (eg, drug tablet return, laboratory tests)
NA		11d	Relevant concomitant care and interventions that are permitted or prohibited during the trial
Y	Outcomes	12	Primary, secondary, and other outcomes, including the specific measurement variable (eg, systolic blood pressure), analysis metric (eg, change from baseline, final value, time to event), method of aggregation (eg, median, proportion), and time point for each outcome. Explanation of the clinical relevance of chosen efficacy and harm outcomes is strongly recommended
Y	Participant timeline	13	Time schedule of enrolment, interventions (including any run-ins and washouts), assessments, and visits for participants. A schematic diagram is highly recommended (see Figure)

For peer review only - http://bmjopen.bmj.com/site/about/guidelines.xhtml

Y	Sample size	14	Estimated number of participants needed to achieve study objectives and how it was determined, including clinical and statistical assumptions supporting any sample size calculations
Y	Recruitment	15	Strategies for achieving adequate participant enrolment to reach target sample size
	**Methods: Assignment of interventions (for controlled trials)**		
	Allocation:		
Y	Sequence generation	16a	Method of generating the allocation sequence (eg, computer-generated random numbers), and list of any factors for stratification. To reduce predictability of a random sequence, details of any planned restriction (eg, blocking) should be provided in a separate document that is unavailable to those who enrol participants or assign interventions
NA	Allocation concealment mechanism	16b	Mechanism of implementing the allocation sequence (eg, central telephone; sequentially numbered, opaque, sealed envelopes), describing any steps to conceal the sequence until interventions are assigned
Y	Implementation	16c	Who will generate the allocation sequence, who will enrol participants, and who will assign participants to interventions
NA	Blinding (masking)	17a	Who will be blinded after assignment to interventions (eg, trial participants, care providers, outcome assessors, data analysts), and how
NA		17b	If blinded, circumstances under which unblinding is permissible, and procedure for revealing a participant's allocated intervention during the trial
	**Methods: Data collection, management, and analysis**		
Y	Data collection methods	18a	Plans for assessment and collection of outcome, baseline, and other trial data, including any related processes to promote data quality (eg, duplicate measurements, training of assessors) and a description of study instruments (eg, questionnaires, laboratory tests) along with their reliability and validity, if known. Reference to where data collection forms can be found, if not in the protocol

For peer review only - http://bmjopen.bmj.com/site/about/guidelines.xhtml

NA		18b	Plans to promote participant retention and complete follow-up, including list of any outcome data to be collected for participants who discontinue or deviate from intervention protocols
NA	Data management	19	Plans for data entry, coding, security, and storage, including any related processes to promote data quality (eg, double data entry; range checks for data values). Reference to where details of data management procedures can be found, if not in the protocol
Y	Statistical methods	20a	Statistical methods for analysing primary and secondary outcomes. Reference to where other details of the statistical analysis plan can be found, if not in the protocol
NA		20b	Methods for any additional analyses (eg, subgroup and adjusted analyses)
NA		20c	Definition of analysis population relating to protocol non-adherence (eg, as randomised analysis), and any statistical methods to handle missing data (eg, multiple imputation)
	**Methods: Monitoring**		
NA	Data monitoring	21a	Composition of data monitoring committee (DMC); summary of its role and reporting structure; statement of whether it is independent from the sponsor and competing interests; and reference to where further details about its charter can be found, if not in the protocol. Alternatively, an explanation of why a DMC is not needed
NA		21b	Description of any interim analyses and stopping guidelines, including who will have access to these interim results and make the final decision to terminate the trial
NA	Harms	22	Plans for collecting, assessing, reporting, and managing solicited and spontaneously reported adverse events and other unintended effects of trial interventions or trial conduct
NA	Auditing	23	Frequency and procedures for auditing trial conduct, if any, and whether the process will be independent from investigators and the sponsor
	**Ethics and dissemination**		

Y	Research ethics approval	24	Plans for seeking research ethics committee/institutional review board (REC/IRB) approval
NA	Protocol amendments	25	Plans for communicating important protocol modifications (eg, changes to eligibility criteria, outcomes, analyses) to relevant parties (eg, investigators, REC/IRBs, trial participants, trial registries, journals, regulators)
Y	Consent or assent	26a	Who will obtain informed consent or assent from potential trial participants or authorised surrogates, and how (see Item 32)
NA		26b	Additional consent provisions for collection and use of participant data and biological specimens in ancillary studies, if applicable
NA	Confidentiality	27	How personal information about potential and enrolled participants will be collected, shared, and maintained in order to protect confidentiality before, during, and after the trial
Y	Declaration of interests	28	Financial and other competing interests for principal investigators for the overall trial and each study site
NA	Access to data	29	Statement of who will have access to the final trial dataset, and disclosure of contractual agreements that limit such access for investigators
NA	Ancillary and post-trial care	30	Provisions, if any, for ancillary and post-trial care, and for compensation to those who suffer harm from trial participation
Y	Dissemination policy	31a	Plans for investigators and sponsor to communicate trial results to participants, healthcare professionals, the public, and other relevant groups (eg, via publication, reporting in results databases, or other data sharing arrangements), including any publication restrictions
Y		31b	Authorship eligibility guidelines and any intended use of professional writers
NA		31c	Plans, if any, for granting public access to the full protocol, participant-level dataset, and statistical code
	**Appendices**		

For peer review only

NA	Informed consent materials	32	Model consent form and other related documentation given to participants and authorised surrogates
NA	Biological specimens	33	Plans for collection, laboratory evaluation, and storage of biological specimens for genetic or molecular analysis in the current trial and for future use in ancillary studies, if applicable

*It is strongly recommended that this checklist be read in conjunction with the SPIRIT 2013 Explanation & Elaboration for important clarification on the items. Amendments to the protocol should be tracked and dated. The SPIRIT checklist is copyrighted by the SPIRIT Group under the Creative Commons "Attribution-NonCommercial-NoDerivs 3.0 Unported" license.

For peer review only
